# Longitudinal single-cell analysis of SARS-CoV-2–reactive B cells uncovers persistence of early-formed, antigen-specific clones

**DOI:** 10.1172/jci.insight.165299

**Published:** 2023-01-10

**Authors:** Lydia Scharf, Hannes Axelsson, Aikaterini Emmanouilidi, Nimitha R. Mathew, Daniel J. Sheward, Susannah Leach, Pauline Isakson, Ilya V. Smirnov, Emelie Marklund, Nicolae Miron, Lars-Magnus Andersson, Magnus Gisslén, Ben Murrell, Anna Lundgren, Mats Bemark, Davide Angeletti

**Affiliations:** 1 Department of Microbiology and Immunology, Institute of Biomedicine, University of Gothenburg, Gothenburg, Sweden.; 2 Department of Microbiology, Tumor and Cell Biology, Karolinska Institutet, Stockholm, Sweden.; 3 Department of Clinical Pharmacology,; 4 Department of Clinical Immunology and Transfusion Medicine, and; 5 Department of Infectious Diseases, Sahlgrenska University Hospital, Region Västra Götaland, Gothenburg, Sweden.; 6 Department of Infectious Diseases, Institute of Biomedicine, University of Gothenburg, Gothenburg, Sweden.

**Keywords:** COVID-19, Immunology, Adaptive immunity

## Abstract

Understanding persistence and evolution of B cell clones after COVID-19 infection and vaccination is crucial for predicting responses against emerging viral variants and optimizing vaccines. Here, we collected longitudinal samples from patients with severe COVID-19 every third to seventh day during hospitalization and every third month after recovery. We profiled their antigen-specific immune cell dynamics by combining single-cell RNA-Seq, Cellular Indexing of Transcriptomes and Epitopes by Sequencing (CITE-Seq), and B cell receptor–Seq (BCR-Seq) with oligo-tagged antigen baits. While the proportion of Spike receptor binding domain–specific memory B cells (MBC) increased from 3 months after infection, the other Spike- and Nucleocapsid-specific B cells remained constant. All patients showed ongoing class switching and sustained affinity maturation of antigen-specific cells, and affinity maturation was not significantly increased early after vaccine. B cell analysis revealed a polyclonal response with limited clonal expansion; nevertheless, some clones detected during hospitalization, as plasmablasts, persisted for up to 1 year, as MBC. Monoclonal antibodies derived from persistent B cell families increased their binding and neutralization breadth and started recognizing viral variants by 3 months after infection. Overall, our findings provide important insights into the clonal evolution and dynamics of antigen-specific B cell responses in longitudinally sampled patients infected with COVID-19.

## Graphical abstract

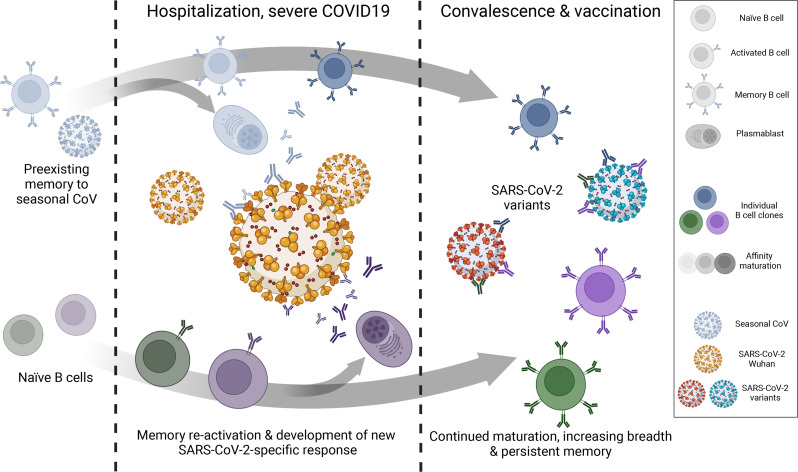

## Introduction

Since the emergence of SARS-CoV-2 in December 2019, there have been over 630 million cases and at least 6.5 million deaths worldwide (1, 2). Despite an extensive vaccination campaign, which substantially reduced morbidity and mortality, the virus is still in circulation, mainly due to the appearance of viral variants that escape preexisting immunity.

Several research groups have described the early immune response upon infection (3–6). During severe infection, general lymphopenia is associated with an increased number of circulating plasmablasts (3), Th1-like CD8 and CD4 cells (5), megakaryocytes, and erythroid cells (7). In serum, Spike-binding (S-binding), neutralizing Abs of the IgA and IgG isotypes emerge early after COVID-19 infection, even before IgM, as reported in some studies (8). Furthermore, it has been suggested that the early plasmablast burst originates from the reactivation of memory B cells (MBC), specific for seasonal Beta coronaviruses (i.e., HKU1 and OC43) (9–14).

With the emergence of viral variants, there has been great emphasis in studying MBC. Early studies with influenza, dengue, and other viral infections in animal models suggest that the MBC pool has greater breadth of antigenic binding, as compared with the plasmablast response (15–18). This led to the hypothesis that, while plasma cells and the serum Abs they produce protect against reinfection with the same strain, the MBC pool represents a diverse reservoir that is able to protect against possible emerging variants. Several studies have now followed MBC development after SARS-CoV-2 infection and reported a continuous increase of B cell receptor (BCR) mutations, consistent with antigen persistence and ongoing germinal center (GC) activity (12, 19–23). The increased number of mutations was also linked with increased affinity and, importantly, neutralization breadth. Interestingly, during influenza infection in animal models, GC persistence has been observed for over 180 days, suggesting this to be a common feature of acute viral infections (24, 25).

mAbs cloned from patients with COVID-19 at different time points after infection demonstrated increased neutralizing breadth against viral variants, even from mAbs belonging to the same clonal family (21, 22). Other studies investigated BCR characteristics during disease (26, 27). Finally, work from the Wilson’s lab linked transcriptional program of single B cell with VDJ properties and antigen specificity, in a cross-sectional cohort (9). However, no study followed the same patients longitudinally during hospitalization, after recovery, and upon vaccination to investigate immune responses, BCR characteristics, and antigen specificity.

To address this, 6 patients with COVID-19 were recruited at hospital admission and were followed during disease and after recovery, for up to 1 year. Half of the patients were also vaccinated by the last time point. We analyzed total peripheral blood mononuclear cells (PBMCs) and B cells using single-cell transcriptomics, expression of 138 surface proteins, antigen binding (S, Receptor Binding Domain [RBD], or Nucleocapsid [N]), and BCR sequences at each of the analyzed time points. Our longitudinal approach allowed us to deduct the origin of antigen-specific B cells and their evolution within each patient. Furthermore, by expressing persisting clones as mAbs, we demonstrate that such clones can be detected within 3 days after hospital admission, persist up to 1 year, and progressively increase their neutralization breadth. Overall, our longitudinal study provides important insights into B cell evolution after viral infections.

## Results

### Longitudinal analysis of peripheral immune responses in patients with COVID-19.

**Figure 1:**
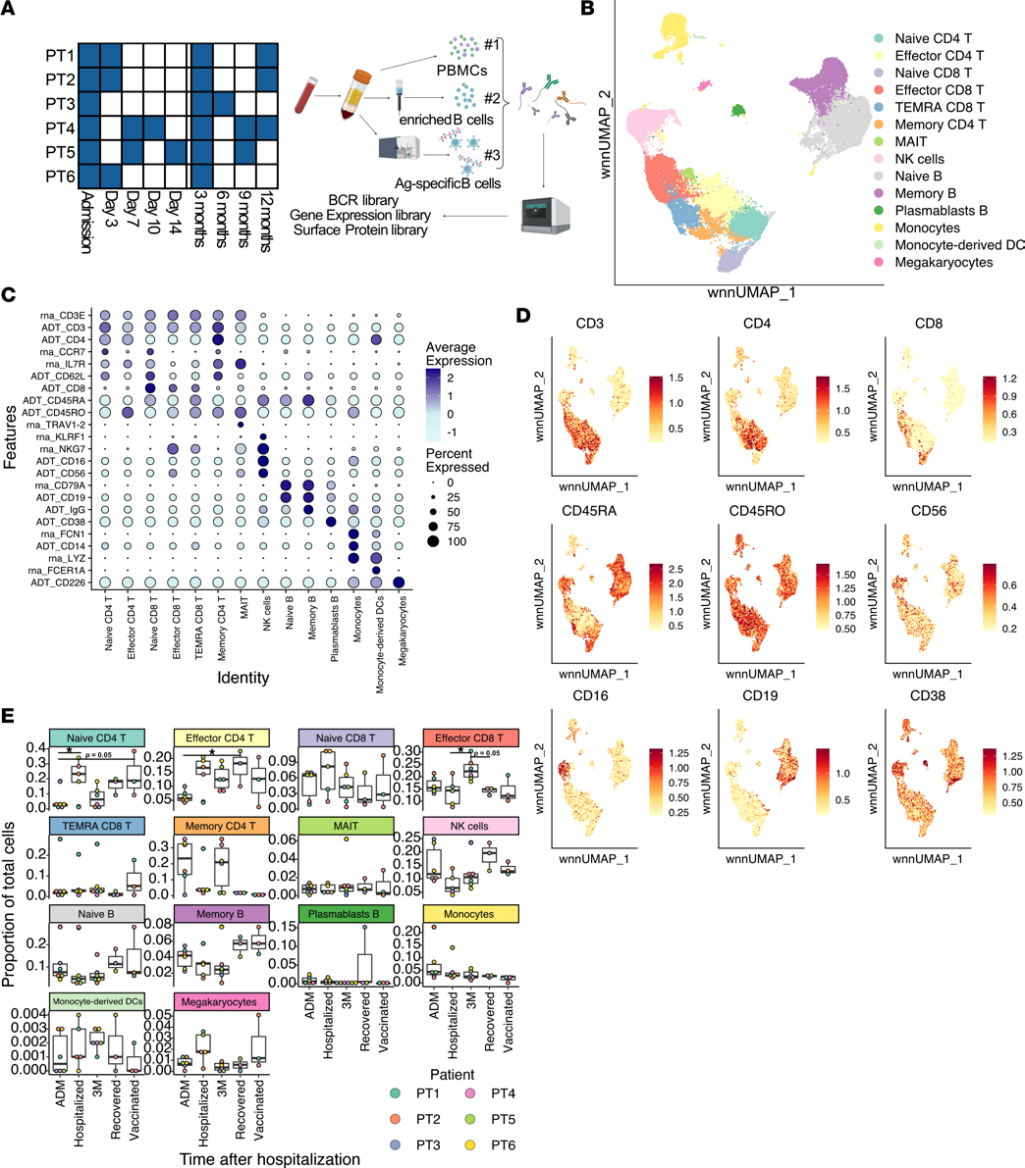
Longitudinal characterization of immune responses in patients with COVID-19. (**A**) Schematic representation of sampling times, indicated in blue, and of the experimental procedure. (**B**) UMAP plot, based on both RNA and surface protein expression, including all 25 samples analyzed. Each dot indicates an individual cell. (**C**) Mean expression of selected marker genes or proteins. Color intensity denotes average expression, whereas dot size is the percentage of cells expressing the gene. “rna” before the gene name indicates gene expression, while “ADT” indicates surface protein. (**D**) UMAP plot showing average expression of selected proteins. Each dot is a cell, and color intensity represents expression. (**E**) Frequency for each of the identified clusters, indicated for each time of sampling and patient. Multiple comparisons were performed using 1-way ANOVA with Tukey’s multiple-comparison test. *P < 0.05.

To better understand the temporal dynamics of the immune response within individual patients, we focused on 6 patients who were admitted to Sahlgrenska University Hospital in June 2020 during the first wave of infections. The patients presented with severe to critical disease and were hospitalized between 5 and 20 days; samples were collected every third to seventh day during this time (Figure 1A and Table 1) (28, 29). All patients returned for a sampling 3 months after hospitalization, and further samples were collected from some patients every third month thereafter. By the 12-month visit in June 2021, the 3 patients who remained in the study had all been vaccinated; patient PT1 had received a first dose of Spikevax (Moderna) 1 week before sampling, patient PT2 had received the first dose of Comirnaty (Pfizer-BioNTech), and patient PT4 had received 2 doses of Vaxzevria (AstraZeneca). PBMCs were prepared and frozen from blood at the time of collection.

To maximize information, each sample was split into 3: total viable PBMCs, a magnetically depleted fraction enriched for B cells, and sorted antigen-specific B cells. Each fraction was labeled with different barcoded hashtag Abs, which allowed for pooling of samples while maintaining the ability to assign each sample and individual (Figure 1A). For enrichment of antigen-specific cells, PBMCs were stained with hashtag Abs and protein-tagged dCODE Klickmers before sorting. Each klickmer featured a unique barcode to allow determination of antigen-specificity and PE to allow flow cytometric sorting. They were loaded with either N, S, or RBD antigen, from the infecting Wuhan strain. To minimize the number of unspecific cells, we also included fluorescently labelled N (BV421) and S (APC) tetramers, and cells were sorted as Live CD19^+^Dextramer^+^ and S^+^ or N^+^ (Supplemental Figure 1A; supplemental material available online with this article; https://doi.org/10.1172/jci.insight.165299DS1). To make sure that only antigen-binding cells would be sorted, initial gates were setup based on Live CD19^+^ IgD^–^Dextramer^+^ and S^+^ or N^+^ (Supplemental Figure 1A). Prepandemic controls showed limited binding to the antigens using this gating strategy (Supplemental Figure 1B). The sorted antigen-specific cells were then pooled with the other 2 fractions and further stained with TotalSeq Human Universal cocktail, a mixture of 138 barcoded surface Abs. Therefore, the transcriptomic, surface antigen phenotype, antigen binding, and BCR sequences could be determined from individual samples.

Strict quality filters were applied, and the average sequencing depth (represented as UMI per cell) was well over 1,000 for all samples, with relatively small differences, indicating good and similar sequence coverage for all samples (Supplemental Figure 2A). Furthermore, the average number of genes expressed per cell was relatively even and, in general, over 1,000, again consistent to good quality of the single-cell RNA-Seq (scRNA-Seq) data (Supplemental Figure 2B). Other quality control (QC) metrics, such as cell complexity and background binding of Cellular Indexing of Transcriptomes and Epitopes by Sequencing (CITE-Seq) Abs, based on 7 isotype controls, were evaluated and demonstrated good quality of our data (Supplemental Figure 2, C and D). Finally, heatmaps of demultiplexed hashtags and protein bait binding, demonstrated specificity of both (Supplemental Figure 2, E and F).

Data were clustered using unsupervised methods based on proteomic and transcriptional data and were visualized in 2D Euclidean space by weighted nearest neighbor–uniform manifold approximation and projection (WNN-UMAP) (Figure 1B and Supplemental Figure 3, A and B). The identity of major cell clusters was then determined based on well-known cell-specific markers (Figure 1, C and D). To define cell type dynamics, we considered only the PBMC pool. Given that not all sampling time points were available for all patients, we grouped them for each patient to allow for proper comparison as follows: admission (ADM), hospitalized (3 day [3D], 7D, 10D, and 14D time points), 3 months (3M) follow up, recovered (6M and 9M time points), and vaccinated (12M time point). The number of total cells varied between samples, due to differences in starting material and recovery, but was still comparable (Supplemental Figure 3C). Time after admission, but not patient sample, impacted the distribution of different cell populations; the frequency of many T cell populations was low at admission, consistent with T cell lymphopenia, but returned to more normal levels already during hospitalization (Figure 1E and Supplemental Figure 3, D and E). The proportion of NK cells was elevated at admission but decreased to lower levels already after 3 days. Furthermore, we detected increased proportions of megakaryocytes during hospitalization, consistent with previous reports (7). Finally, among B cells, plasmablasts were elevated during hospitalization and MBC tended to increase after hospitalization, even when excluding antigen-specific, sorted cells from analysis (Figure 1E). Altogether, the data demonstrate alterations of the circulating immune cell populations during COVID-19 infection that returned to normal levels after recovery.

### Enrichment of circulating Th1 CD4 and cytotoxic CD8 T cells early after disease onset.

**Figure 2:**
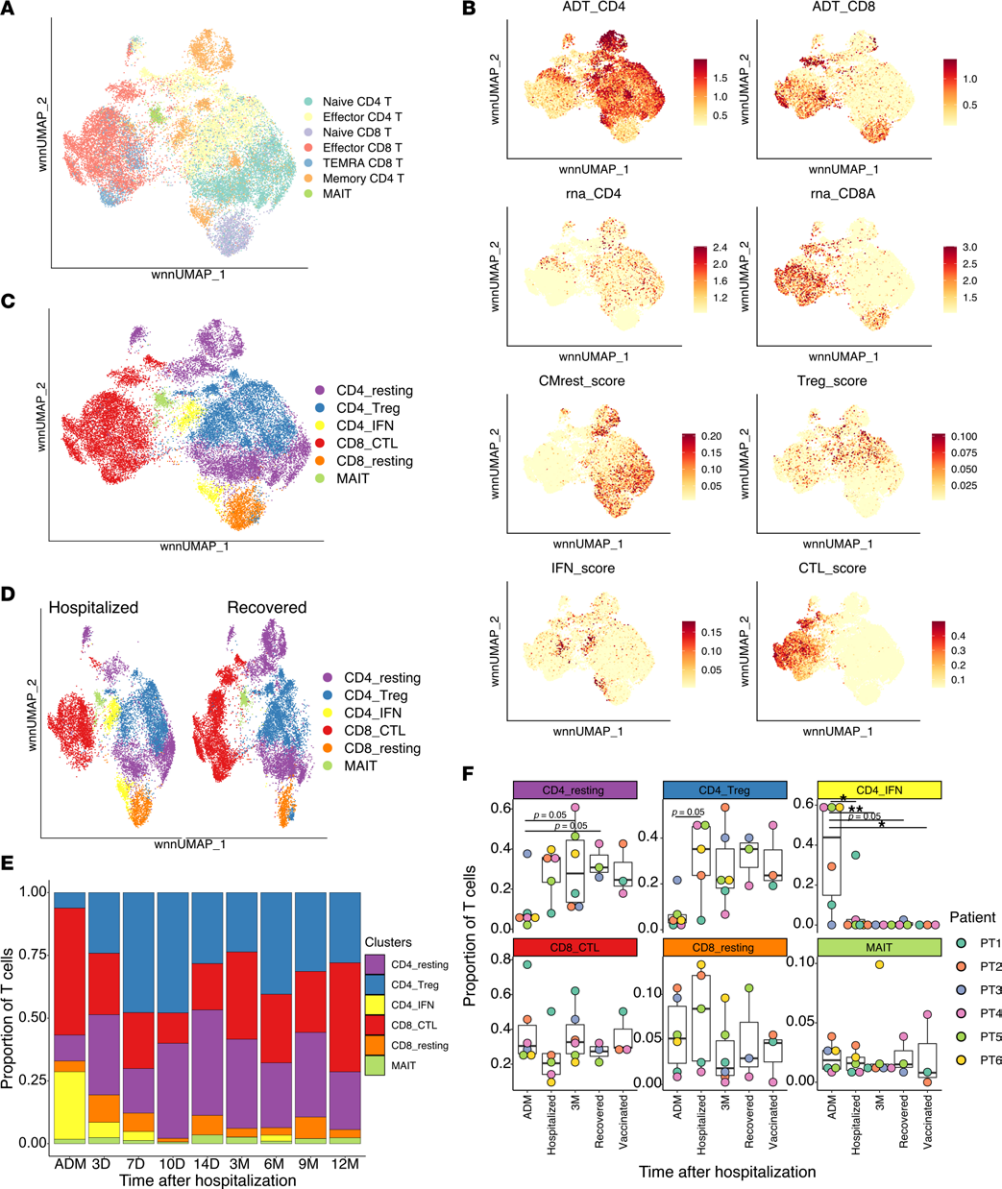
Longitudinal characterization of T cells in patients with COVID-19. (**A**) UMAP plot, based on both RNA and surface protein expression, of T cells. Cluster names are based on Figure 1. Each dot indicates an individual cell. (**B**) UMAP plot showing average expression of selected genes (rna), proteins (ADT), or combined gene signatures, according to ref. 30. “ADT” indicates surface protein expression, while “rna” shows transcript expression. “CMrest” score is genes associated with resting central memory; “Treg” score represents genes associated with Tregs; “IFN” score is genes associated with IFN response; and “CTL” score represents genes associated with cytotoxic T cell responses. Each dot is a cell and color intensity represent expression. (**C**) UMAP plot of T cells as in **A**, but grouping is based on gene signature expression as in **B**. (**D**) UMAP plot as in **C** but split based on hospitalization status. (**E**) Quantification of the proportion of cells for each T cell cluster at each sampling time. All patients were included. (**F**) Frequency for each of the identified clusters, indicated for each patient and time of sampling. Multiple comparisons were performed using 1-way ANOVA, with Tukey’s multiple-comparison test. *P < 0.05; **P < 0.01.

T cells are crucial to limit ongoing intracellular viral infections and to guide B cell responses. To further characterize T cell response, we subsetted the PBMC pool on these populations, reran unsupervised clustering analysis, and visualized it by WNN-UMAP, using combined protein and RNA data (Figure 2A). The different populations clustered similarly as before. However, recovered T cell number was more variable, as compared with total cells (Supplemental Figure 3F). Indeed, this was marked in admission samples and might be due to early T cell lymphopenia. To gain deeper insights into T cell activation and transcriptional states, we used functional gene sets for T cell subpopulations previously described (30). Based on gene set expression, we were able to classify CD8- and CD4-expressing T cells into resting cells, Tregs, IFN-responding cells, and cytotoxic T cells (Figure 2, B and C). Interestingly, we identified 2 clusters of CD4 T cells with a very strong IFN gene signature. The signature included several IRF genes, IFI genes, and other canonical genes associated with IFN response (*MX1*, *STAT1*, *JAK2*). Further analysis revealed the CD4_IFN population to be highly expanded at admission, for all patients, and retained for up to 7 days of hospitalization, when it disappeared (Figure 2, D and E, and Supplemental Figure 3, G and H). One other expanded population at admission was CD8 cytotoxic T cells (CTL), characterized by expression of *GZMK* and *GNLY*, among others (Figure 2B). Approximately 75% of circulating T cells at admission belonged either to CD8 CTLs or IFN-responding CD4 T cells, independently of patient, consistent with a strong antiviral response (Figure 2F). However, their relative levels returned to normal 3–7 days after admission and were then maintained at fairly constant levels for at least 1 year after recovery.

Overall, our data suggest a rapid expansion of antiviral T cells in the circulation upon severe COVID-19 infection. CD4 T cells with an IFN gene signature rapidly increase early after infection and return to normal levels within 3–7 days during nonlethal infection. In addition, CD8 CTLs are also enriched up to 7 days after admission, although in this case, there were larger differences between individuals.

### Switched MBCs exhibit unique transcriptional features.

**Figure 3:**
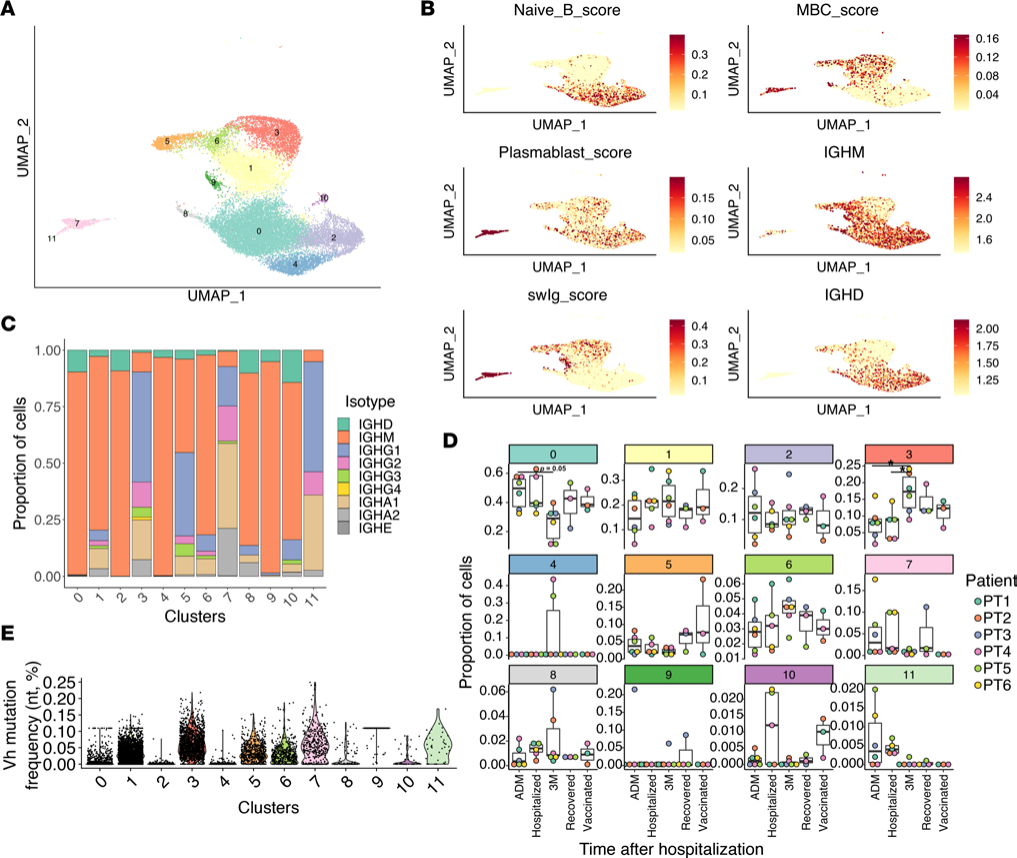
Longitudinal characterization of B cells in patients with COVID-19. (**A**) UMAP plot of B cell populations. Each dot indicates an individual cell. (**B**) UMAP plot showing average expression of selected genes or combined gene signatures, according to ref. 9. Each dot is a cell, and color intensity represents expression. (**C**) Quantification of the proportion of cells for each B cell cluster divided by isotype. All patients were included. (**D**) Frequency for each of the identified clusters, indicated for each time of sampling and patient. Only cells belonging to the PBMC and enriched B cell pools were considered for this analysis. Multiple comparisons were performed using 1-way ANOVA, with Tukey’s multiple-comparison test. *P < 0.05. (**E**) Graph showing VH gene mutation frequency per UMAP clusters as in **A**. Each dot represents an individual cell. Multiple comparisons were performed using 1-way ANOVA with Tukey’s multiple-comparison test. All pairwise comparisons had *P* < 0.0001, except cluster 11 versus clusters 1, 3, 5, 6, and 7; cluster 10 versus clusters 0 and 8*;* cluster 1 versus cluster 6; cluster 2 versus cluster 4; and cluster 3 versus cluster 7, whose comparisons were all nonsignificant.

A key feature of our study is the ability to longitudinally follow antigen-specific B cell clones during hospitalization and after recovery. B cells were analyzed separately from other cell types as above, and upon unsupervised reclustering, 12 B cell clusters were identified (Figure 3A and Supplemental Figure 4, A and B). Recovered B cell numbers were comparable among samples (Supplemental Figure 3I). We used gene signature scores to classify these different populations (9) (Figure 3B). Clusters 0, 2, 4, 8, and 10 had a strong signature associated with naive B cells, which included *BACH2*, *ZBTB16*, *APBB2*, *SPRY1*, *TCL1A*, and *IKZF2*. Conversely, clusters 1, 3, 5, and 6 expressed *CD27*, *CD80*, *CD86*, *TOX*, *TNFRSF13B*, and *FCRL5* — core components of the MBC gene signature that was used. Clusters 7 and 11 expressed *CD38*, *IRF4*, *PRDM1*, and *XBP1*, confirming their differentiation into Ab-secreting plasmablasts. Furthermore, we classified cells by IgM expression or switched immunoglobulin (swIg) signature, which included IgG and IgA expression. Most naive B cells expressed IgM, while the plasmablasts were mostly class switched. Among MBC, we clearly identified clusters 3 and 5 as switched MBC, while cluster 1 was mainly composed of IgM-MBC and cluster 6 of IgD/IgM-MBC (Figure 3, B and C, and Supplemental Figure 4C). The gene expression observations were confirmed when analyzing the isotype from the expressed BCR, using data from the VDJ sequencing (Figure 3C); here, we could observe an IgG bias in the swIg memory population and an IgA bias in the plasmablasts (clusters 7 and 11). It should be noted that VDJ sequencing is not fully able to pick up IgD-expressing B cells, due to the primers used, which explains the low IgD representation in naive clusters depicted in Figure 3C.

While the proportion of cells within most of the naive and MBC clusters were stable over time and between patients, there were significant fluctuations for plasmablast (Figure 3D and Supplemental Figure 3, J and K). Plasmablast clusters 7 and 11 were high at admission and during hospitalization, as previously reported (3), but they returned to low levels after recovery. When considering only the PBMCs and B cell–enriched pools, naive and memory cells were stable throughout the study period (Figure 3D). When only the antigen-specific sorted fraction was analyzed, antigen-specific cells were clearly enriched in the swIg memory clusters 3 and 5 and increased over time, consistent with constant new memory cell generation (Supplemental Figure 4D).

Mutation frequencies of the different B cell populations, regardless of antigen specificity, indicate that plasmablasts have higher mutation rates than other cell types, despite appearing earlier after hospitalization (Figure 3E). These data are consistent with plasmablasts deriving from rapid differentiation of preexisting MBC, specific for seasonal CoV, as previously suggested (9–11). In agreement with our gene expression characterization, MBC clusters were mutated, while naive B cell clusters were not. Overall, this analysis demonstrates that antigen-specific switched MBC increase over time, while IgM-MBC remained stable throughout the study period.

To gain better insights into differences between these 2 MBC subpopulations, we compared gene expression between swIg-MBC (clusters 3 and 5) and IgM-MBC (cluster 1). The differential gene expression analysis revealed significant differences in expressed genes between the 2 (Supplemental Figure 4E), as previously described using total RNA-Seq analysis (31). Genes upregulated in swIg-MBC included integrins *ITGB1* and *ITGB2*, IFN-induced *IFI30*, and genes such as *CD86*, *TNFRSF1B*, and several HLA genes, overall suggesting a heightened activation status. IgM MBC also expressed survival genes, like *MYC*/*BTG1* and *FOXP1*, as well as the chemokine receptors *CCR7* and *CXCR4*. Gene ontology (GO) analysis of upregulated genes revealed a much stronger activation profile for swIg-MBC, which included signs of increased signaling, cytokine responses, proliferation, response to IFN, antigen presentation, and more (Supplemental Figure 4F). Overall, the data suggest that swIg-MBC is in a more activated state, and possibly has a lower reactivation threshold upon reinfection.

### B cells demonstrate differential dynamics depending on antigen specificity.

**Figure 4:**
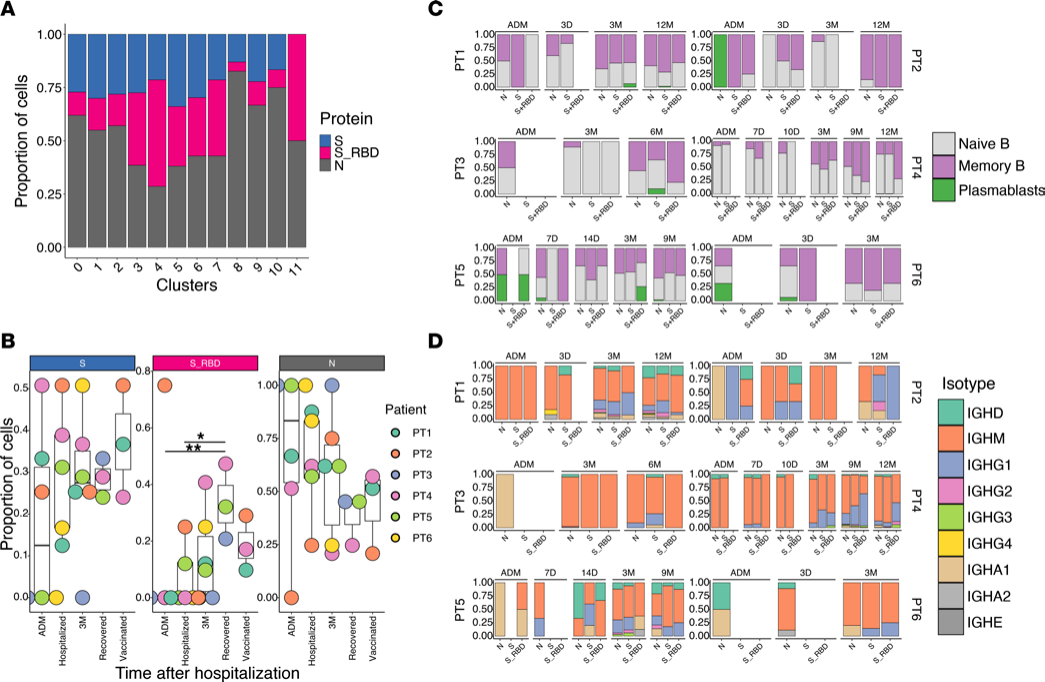
Longitudinal characteristics of antigen-specific B cells. (**A**) Quantification of the proportion of protein-binding cells for each B cell cluster. N is nucleocapsid, S_RBD is receptor binding domain and Spike binders, while S is Spike non-RBD binders. (**B**) Frequency for each protein specificity, indicated for each time of sampling and patient. Multiple comparisons were performed using 1-way ANOVA, with Tukey’s multiple-comparison test. *P < 0.05; **P < 0.01 (**C**) Quantification of the proportion of B cell subtypes for each protein-binding specificity, indicated for each patient and sampling time. (**D**) Quantification of the proportion of B cell isotype for each protein-binding specificity, indicated for each patient and sampling time.

To dissect the evolution of antigen-specific B cell subtypes, we analyzed the antigen specificity of B cells clusters. This was assessed by looking at unique protein barcodes for protein binding cells, which were indeed highly enriched in the sorted population (Supplemental Figure 4G), as expected. In the following section, cells binding to both RBD and S will be defined as S_RBD, while cells binding to S but not RBD will be defined as S. The MBC subset were binding equally to S and to N, with swIg-MBC (clusters 3 and 5) exhibiting a preference for RBD (Figure 4A). While binding was generally lower, antigen binding could also be detected among naive B cells. These could be broadly defined into 3 groups: clusters 0 and 2 were approximately 50% S and 50% N binding; cluster 4 was mainly composed of S, especially RBD binders; and, finally, all other naive B cell clusters had a majority of N-binding cells (Figure 4A). 

Given the difference in specificity between B cell subpopulations, we decided to investigate the dynamics of antigen-specific cells over time (Figure 4, B and C). We found that, among MBC, the proportion of RBD-specific cells continues to increase with time, even in the absence of vaccination. S non-RBD–binding MBC had a more complex pattern; their relative frequency decreased during hospitalization but rebound to admission levels by 3 months after infection and were also slightly boosted upon vaccination (12-month time point). In contrast, N-specific MBC were mostly stable throughout the study period.

Longitudinal analysis of antigen specificity and isotypes further highlighted differences between populations (Figure 4D). At admission, we could detect a relatively large number of N-specific IgA cells, and this number correlated with a higher proportion of plasmablasts. At later time points, most patients developed an antigen-specific IgG response, concomitant with an increased proportion of antigen-specific MBC, which was slightly more marked for S- and RBD-specific cells as compared with N-specific cells. Vaccine administration was particularly efficient in increasing the proportion of isotype-switched S- and RBD-specific cells in 1 patient (PT2) but did not have a strong effect in the other 2 vaccinated patients.

We could track the longitudinal dynamics of antigen-specific B cells over time. We found that switched RBD-binding MBC increased over time, peaking by 9 months. In contrast, S, non-RBD, and biding MBC were mostly stable.

### Antigen-specific B cells possess unique repertoire features.

**Figure 5:**
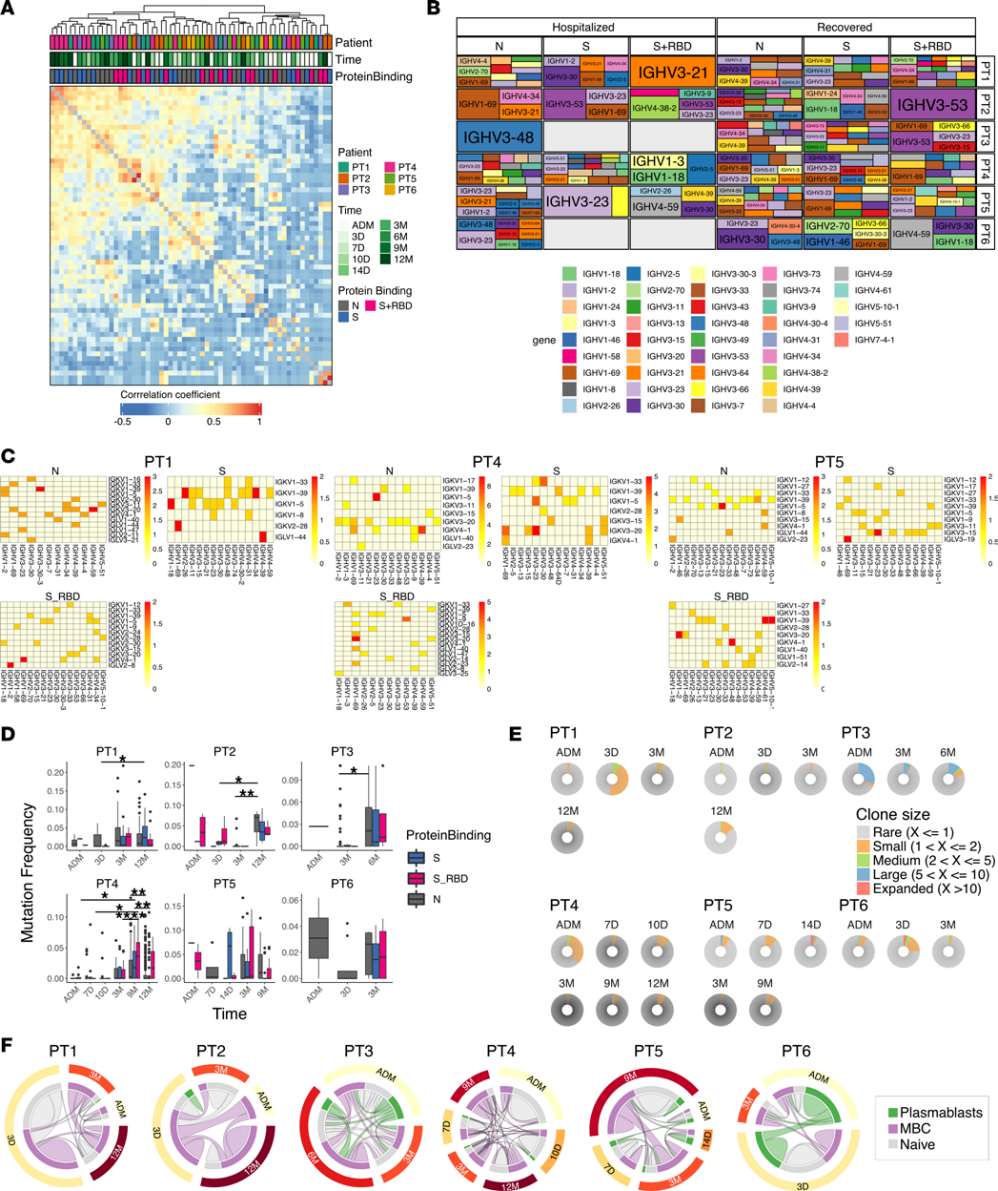
Antigen-binding B cells are selected and persist from infection up to 1 year. (**A**) Hierarchical clustering of Pearson’s correlation of the V gene repertoire. Each tile represents the correlation of the V gene repertoire. Color intensity indicates correlation strength. (**B**) Tile plot indicating the 10 most frequent V genes used per patient, along with protein binding and hospitalization status. Size of the tile is proportional to the repertoire space occupied. (**C**) Heatmaps showing the frequency of each patient’s heavy- and light-chain gene pairings for B cells binding the indicated antigens. (**D**) Graph showing VH gene mutation frequency indicated for each patient, time of sampling, and antigen binding. Data are presented as median and interquartile range. Multiple comparisons were performed using 2-way ANOVA with Bonferroni correction for multiple comparisons. *P < 0.05, **P < 0.01, ****P < 0.0001 (**E**) Pie chart showing B cell clonal expansion indicated for each patient and time point. B cells were binned into rare clones (1 member), small (2 members), medium (between 3 and 5 members), large (between 6 and 10 members), and expanded (over 11 members). (**F**) Circos plot showing clonal relationship within each patient at different sampling times, for clonal families with at least 2 members. Connecting lines indicate shared clones, and the size of the circle and connector is proportional to the repertoire space occupied. Outer circle indicates sample time, while inner circle and connectors are colored depending on cell type.

To determine whether antigen-specific cells were indeed enriched for distinct Ab gene-usage signatures, we first used a Pearson’s correlation matrix to analyze variable heavy-chain (VH) gene–usage overlap (32). As a control, the analysis was also performed just taking into account the patient and sampling time point, and given the mostly private nature of V gene repertoire (33), samples clustered mainly by patient, regardless of sampling time (Supplemental Figure 5A), as expected. However, when we grouped patients — not only by time, but also by antigen binding — the picture was different (Figure 5A); with the exception of PT4, which maintained a strong private VH signature, the clustering was mainly dictated by sampling time and antigen specificity. This suggests that antigen specificity is a strong determinant for VH selection and that VH gene usage in antigen-specific B cells was different during hospitalization as compared with after recovery. This could be explained by the fact that early responses potentially originate from cross-reactive MBC, while later B cells are mainly novel MBC that have matured within the GC.

To better visualize VH gene usage, we generated tile plots, where, for each antigen, the top 10 VH genes were represented proportionally. By grouping by hospitalization status and patient (Figure 5B), we were able to dissect similarities and differences between individuals and proteins. For S non-RBD, it has been previously reported that VH gene usage is skewed toward VH1-24 (N terminal domains [NTD]), VH3-30 (S2), and VH3-33 in convalescence (34–41). We found that all patients use such genes but in different proportions; for instance, PT2 has strong preference for VH1-24 and VH3-33, while PT4 mainly utilizes VH3-30. Importantly did not find such a strong bias during early response, where only patient PT1 has strong VH3-30 preference, which is lost after recovery. Similarly, VH3-23 and VH4-39 have been previously described for N-specific B cells. We did, indeed, find a similar bias in recovered samples, with some patients being more prone to using VH4-39, while others use VH3-23. Here the VH3-23 bias was present in 3 of the 6 patients at an early time point, but VH4-39 usage was detected only in PT4, again arguing for an evolving B cell landscape. For RBD, several VH genes have been associated with potently neutralizing mAbs (40, 42, 43), and we detected some of those in our patients (VH3-23, VH3-30, VH1-69, VH3-53), again with marked difference between samples collected during hospitalization and recovery.

Similarly, patient-specific heavy-light chain pairing confirmed that the preference of certain V gene pairings was driven by antigen (Figure 5C and Supplemental Figure 5B). Most patients had VH3-53 among the top pairings for RBD reactive Abs; however, the light-chain pairing was different between patients. PT1 paired VH3-53 in RBD-reactive Abs with Vk3-20, while PT4 paired with Vk1-9. Both these pairings have been already described (40). Recently, a similar mAb, pairing VH3-53 with Vk3-20 has been described as a broad neutralizer against viral variants and belonging to the RBD-2 cluster (44). Overall, we detect some similarities between individuals with both time after infection and protein binding being major drivers of VH gene usage.

### Limited clonal expansion and continuous evolution of antigen-specific B cells.

Next, we wanted to assess when antigen-specific clones arose and how long they persisted in the memory pool. By sampling longitudinally, we were able to follow the clonal expansion and evolution of antigen-specific cells during hospitalization, after recovery, and upon vaccination. Mutation rate stratified by antigen binding was steadily increasing over time in all patients, regardless of antigen specificity (Figure 5D and Supplemental Figure 5C). In contrast, the overall mutation rates of B cells were constant throughout the study period (Supplemental Figure 5D). Our observation is consistent with previous data suggesting persistent GC after COVID-19 infection and continued evolution of B cells (12, 19–22). Apart for PT2, we did not detect a significant increase in somatic hypermutation (SHM) after vaccination.

Surprisingly, we observed a very low level of clonal expansion (Figure 5E), even when considering only protein binders (Supplemental Figure 5E). Except for PT3, which had 1 longitudinal and highly expanded clone, most other patients did not show signs of substantially expanded B cells clones, either early or late. Even during recovery and after vaccination, we could not measure substantial clonal expansion (Figure 5E). Clonal relationship analysis demonstrated mostly unique clones and few persistent clones across the study period (Supplemental Figure 5F). To better visualize clones that were maintained over time, we focused on clonal families with more than 1 member (Figure 5F). Clonal analysis on this subset of B cells revealed that most patients maintained at least some clones, which were generated during infection, in circulation for up to 1 year (Figure 5F). Importantly, clones related to early plasmablasts and/or early MBC were found to persist for up to 1 year in the majority of patients (Figure 5F and Supplemental Table 1). However, these were not substantially expanded upon vaccination. These results indicate that most of the stable memory clones are indeed formed at an early stage after infection. When considering antigen specificity, we found that it was easier to assign antigen-binder status at later time points, even for clones within the same family. We reasoned that SHM and affinity maturation might play a role in this phenomenon, with cells at early time points being of too low affinity to bind to the recombinant protein used for sorting.

The data presented here suggest a limited clonal B cell expansion after COVID-19 infection but demonstrate the early formation, persistence, and evolution of individual clones for up to 1 year after infection.

### B cells increase antigen binding and neutralizing breadth over time.

**Figure 6:**
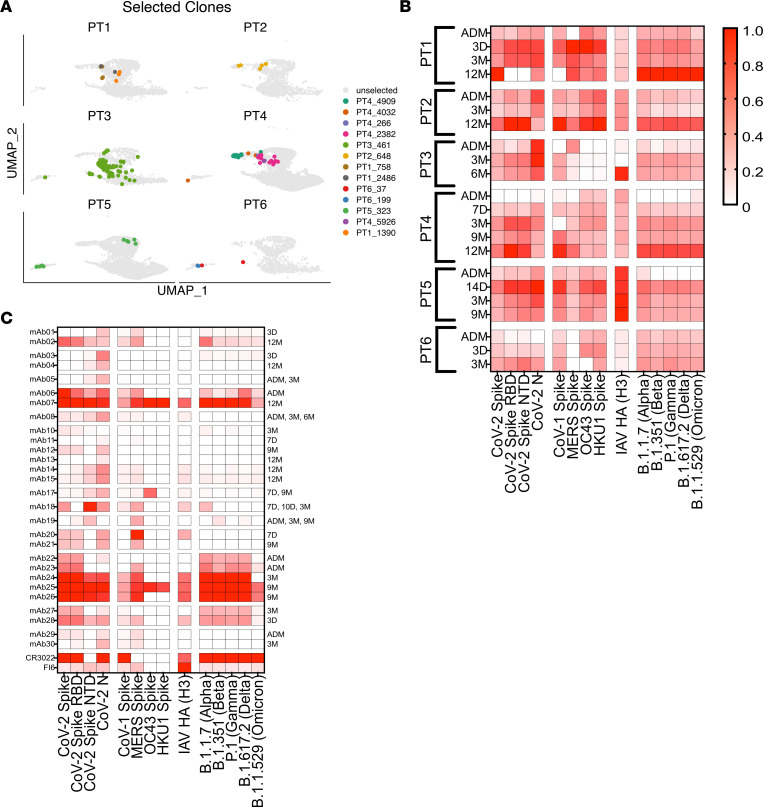
Continued evolution of serum antibodies and B cell responses within patients. (**A**) UMAP plot of B cell clusters highlighting selected persistent clones per patient. (**B** and **C**) Serum Ab (**B**) and mAb (**C**) binding to a panel of viral antigens, as detected by an electrochemiluminescent binding assay. Color intensity indicates the normalized log binding intensity, where the top binder is set to 1.

To further investigate how affinity maturation can influence B cell evolution and specificity, within the same patient, we expressed 30 mAbs derived from all 6 patients, belonging to 13 clonal families that were found at early and late time points within the same patient (Figure 6A and Supplemental Table 1). These included 5 mAbs that were found to be identical at early and late time points. Two mAbs (mAbs 9 and 16) failed to be expressed.

First, we tested all available sera (Figure 6B) and mAbs (Figure 6C) for binding to a panel of viral antigens, using a multiplex electrochemiluminescence–based assay. The first panel included full-length S, as well as RBD and NTD of S and N from the SARS-CoV-2 Wuhan strain; S from SARS-CoV-1, MERS, HKU-1, and OC43; and hemagglutinin (HA) from Influenza A virus H3/Hong Kong. The second panel included S proteins from several SARS-CoV-2 variant strains: Wuhan, Alpha (B.1.1.7), Beta (B.1.351), Gamma (P.1), Delta (B.1.617.2, AY.4, and AY.4.2), and Omicron (B.1.1.529 BA.1). Importantly, all our patients were hospitalized in June 2020 and, thus, were infected with the ancestral strain. The serum Ab binding profile was similar for all patients and showed fairly strong binding to most of the antigens. Sera collected at admission was also reactive to seasonal CoV for 5 of 6 patients (Figure 6B). SARS-CoV-2–specific IgG developed within few days reached a peak by 3 months in most patients, and levels were then relatively stable throughout the study period. We could detect a sharp increase in IgG Abs binding to all S but not N or control influenza antigens after vaccination (12-month time point; Figure 6A). By comparing this with the B cell data, we can speculate that, after vaccination, many MBC directly differentiate into Ab-secreting plasma cells.

The majority of the expressed mAbs was able to bind at least 1 of the tested proteins, confirming the specificity of our sorting approach (Figure 6C). In general, binding to N was harder to assign, as several strong S-specific mAbs also showed N signal, including the CR3022 Ab used as a binding reference (45). Therefore, we assigned N specificity only to mAbs with stronger N signal, as compared with other proteins. Four mAbs were specific for the N protein (mAbs 3, 4, 5, and 17). mAb18, which was identical at early and late time points, was specific for NTD of S. This specific BCR, of the IgA1 isotype, was present at the 7-day, 10-day, and 3-month sampling time points. This mAb did not cross react with seasonal CoV but had some minor cross-reactivity with MERS-S and Alpha-S. For family PT4_2382 (mAbs 10–15), we could not confidently assign a protein specificity. For S-specific cells, the large majority of cloned mAbs were RBD directed. Importantly, we could detect that cells, clonally related to the early plasmablasts, reemerged at later time points as MBC. This was the case for one of our clonal families (with mAbs 22–26), belonging to patient PT5. Here mAbs 22 and 23 were identified at admission from plasmablasts, showing strong RBD binding and an already high mutation rate of 7%–8%. At the 3- and 9-month follow-up, clonal relatives were then identified in the MBC pool with an increased mutation rate of 7%–11%. Importantly, mAbs from the memory pool (mAbs 24–26) showed significantly higher antigen binding breadth, including some binding to Omicron BA.1. All of these mAbs were originally IgA1. The data here suggest that (a) clonal relatives of early plasmablasts also entered the GC and, there, continued to evolve as MBC with further binding breadth, or (b) the same GC clones can generate early plasmablasts and late MBC. When tested on the S variant panel, we analyzed the response for every clonal family. Apart from the pair mAbs 20 and 21, most S-specific families consistently increased breadth over time, regardless of vaccination status. While most families eventually acquired binding to all variants, including Beta and Delta, binding to Omicron BA.1 was not obtained by all and was, generally, of lower magnitude. Specificity of the highly sensitive electrochemiluminescence approach was confirmed using traditional ELISAs. As expected, some interactions fell below detection in this case, giving less background, yet the ELISA in general corroborated our the results and demonstrated a level of polyreactivity for some of the mAbs (Supplemental Figure 6, A and B).

To verify whether the increase of binding breadth correlated with increased neutralizing activity against viral variants, we tested the mAbs using HEK293T-ACE2 cells and SARS-CoV-2 pseudotyped lentivirus (46). Of the S-binding families, mAb01, mAb02, mAb27, and mAb28 did not neutralize, despite an increase in binding breadth (Figure 6C and Supplemental Figure 6C). mAb06 neutralized poorly, but its later relative mAb07 was potent against ancestral SARS-CoV2 S, as well as against Alpha, Beta, Gamma, and Delta (Supplemental Figure 6, C–E). The family from which we had most binding clones, PT5_323, showed increasing neutralization breadth. Of the mAbs isolated at admission, mAb22 did not neutralize, while mAb23 neutralized poorly against ancestral S. The 3-month relative, mAb24, had higher IC_50_ against ancestral S, similar neutralization capacity against Alpha, and some weak neutralization against Beta, Gamma, and Delta. The 9-month mAbs, mAb25 and mAb26, were potent against all strains, except Omicron BA.1 (Supplemental Figure 6, C–E).

In summary, we demonstrate a continued evolution of B cells, irrespective of vaccination, with B cells acquiring increased antigen binding and neutralization breadth over time.

## Discussion

Despite a successful vaccination campaign, SARS-CoV-2 is still circulating and causing significant disease (1). Viral variants have continued to emerge (47–50), and there is a need to better understand the development of B cell immunity after infection and vaccination. While levels of neutralizing Abs in sera are a good correlate of protection from infection, circulating MBC, with varying specificity and breadth, can also be rapidly reactivated by reinfection (51, 52). Even if the specific B cells are not able to protect from infection, MBC can rapidly differentiate to plasmablasts or reenter GCs, thus fighting the virus, limiting viral replication, and decreasing disease severity. Many studies have followed serum Ab activity in patients longitudinally, while less is known about the dynamics and specificity of MBC during disease and convalescence (53–56). A few studies have analyzed the clonal relationship of B cells, but these usually start with samples collected 1 month after disease onset, at the earliest, excluding any comparisons to acute plasmablast responses (12, 19–22). A study by Dugan et al. used a technical setup similar to ours; however, the majority of the patients were not followed longitudinally but rather sampled cross-sectionally, either during hospitalization or after recovery (9). To the best of our knowledge, a detailed study where the transcriptional dynamics of total PBMCs and B cells followed longitudinally during disease, convalescence, and after vaccination in individual patients has not previously been performed. This setting allowed us to track responses in few but densely sampled individuals to elucidate the dynamics of antigen-specific B cells, as well as more general immune responses, after COVID-19 infection.

In our study, we analyzed samples from 6 patients with severe/critical COVID-19 infection, and we report significant alterations in the circulating immune populations during disease. As described previously (5), we demonstrate the presence of CD4 T cells with a strong IFN signature early during hospitalization that disappeared by 3 months. Other significant alterations in the immune cell composition or transcriptional features normalized by day 14 after admission. Given the low number of patients, it is not possible to make any solid conclusion regarding the relationship between the analyzed immune responses, comorbidities, and general immune status. Three of our patients (PT2, PT3, and PT4) had respiratory comorbidities; however, they did not have any immediately noticeable difference in the frequency of the different immune cell populations or B cells. PT2 had partial IgA deficiency (due to anti-IgA IgGs) and decreased IgG2 serum levels; however, the frequency of MBC and antigen-specific MBC were normal and even higher than others. In general, we can conclude that, regardless of comorbidities, immune-status, age, and sex, immune responses showed similar features in all patients.

The main goal of the study was to investigate longitudinal B cell clonal dynamics and antigen specificity. Here, we reveal that, upon infection, patients do not present largely expanded B cell clones in the blood. Rather, several smaller clones can be detected at all sampled time points, even among early plasmablasts, and these do persist in circulation for at least 1 year, even after vaccination. Furthermore, mAbs within the same patient, belonging to the same clonal family, acquired increased binding to several viral variants over time, revealing persistent ongoing clonal affinity maturation.

A significant observation is the expansion of MBC after recovery, with an increased frequency of antigen-specific B cells and higher SHM across binders. However, this was not significantly accelerated by vaccination. Indeed, the SHM rate, in antigen-specific cells, was similar between 9 months after infection and 12 months (which was after administration of 1 or 2 vaccine doses). This suggests that, in these patients, vaccination was not sufficient to elicit a strong clonal expansion and reactivation of infection-derived MBC, at least when sampled from the circulating PBMCs. In contrast, data from sera indicate a strong differentiation of MBC to Ab-secreting plasmablasts in response to vaccination. We could not detect antigen binding for the majority of plasmablasts due to their low surface Ig expression, but most of those we could identify were RBD specific. This, combined with the already high mutation rate and the predominant IgA and IgG isotype expression, confirms that these most likely originate from seasonal CoV-specific MBC, which were rapidly reactivated (9–11). While many of these plasmablasts did not have clonal relatives at later time points, there were notable exceptions, demonstrating that, after activation, clonal relatives to these early plasmablasts must have entered GC and acquired further mutations and breadth before differentiating to MBC. An alternative, possible explanation is that early plasmablasts were, in fact, generated from naive B cells forming COVID-19–induced GC, although this is not fully compatible with the high mutation rates of these cells.

When comparing IgM and switched MBC for their transcriptional signatures, we detected significant differences. Some of the genes identified in our study are similar to previously identified sequences using bulk RNA-Seq (31). Furthermore, our GO analysis highlighted that swIg-MBC show a more “activated” transcriptional signature as compared with IgM MBC, despite having similar mutation rates. Furthermore, swIg-MBC preferentially bound RBD, while memory IgM had more N-binding. This might imply that RBD-specific swIg-MBC are primed to get reactivated upon reinfection. Increased RBD binding in the MBC population was previously reported (12), but we add to the data by showing that vaccination does not dramatically increase this targeting.

We were surprised by the limited clonal expansion after disease and vaccination. While previous studies (12, 20) reported similar findings, their first sampling point was approximately a month after infection. A more comprehensive BCR repertoire sequencing also indicated a lack of clonal expansion after mild disease (26) but high clonal expansion starting a month after vaccination. Our data show that individuals in our study had a more diverse response after vaccination, given their preexisting Ab immunity, as recently suggested in another study (57). Despite this, we could identify a number of antigen-specific and persistent clones together with the emergence of novel, antigen-binding B cells at later time points. Importantly, like essentially all single-cell studies, our work has limited sampling depth of the BCR repertoire (58). This limits some of the conclusion we can make from our analyses but makes it even more remarkable that we were still able find persisting clones, even within this limited repertoire space. It is possible that more persisting clones would be revealed, had the sequencing depth been greater. While clonal expansion is undoubtedly vigorous in animal models upon vaccination or viral infection, the situation in humans is clearly more complex. Animal studies suggest that GC have the ability to maintain a diverse response (59, 60), but how this is attained is not completely clear. With B cell activation after COVID-19 infection initially consisting of recalled seasonal CoV-specific B cells, it could be that such B cells, in combination with preexisting serum Abs, could promote the diversity of the ongoing GC. A more diverse GC would favor novel responses and the ability of the B cells to adapt to existing and future viral variants (15, 17, 61).

The main limitation of this study is the relatively low number of analyzed patients. This makes it difficult to link some of the gene expression and cellular data to the clinical status of the patients. However, given the type of analysis performed on the patients and our wish to follow immune responses over time, we preferred to have a smaller cohort of regularly sampled patients rather than a larger number of individuals with less-frequent sampling. Stronger responses may indeed have appeared after a second or third dose of vaccine. Given the variability between individuals, future studies including more patients, especially after vaccination, would be needed to confirm our results. Furthermore, having started our study before the emergence of the occurrence of dominant viral variants, we only included Wuhan-S as our sorting antigen. Inclusion of S proteins from viral variants in the sorting and sequencing procedure would have been a valuable addition to allow for better understanding of future protection in the individuals. Finally, while we demonstrate binding breadth and neutralization capacity of the cloned mAbs, it is impossible to know if this represents their in vivo protective capacity. Future studies will also need to address the presence and persistence of antigen-specific B cells in tissues and their clonal relationship with circulating B cells. A previous study reported persistence of immune cells in organs up to 6 months after COVID-19 infection (62), but their clonality with circulating B cells was not investigated. Fine-needle aspirates of LN (63, 64) could give important insights into this issue, but the optimal — although technically impossible — sampling site would be the lungs, where tissue-resident BMEM might be the first line of protection against viral reinfection.

Overall, we show a number of persisting, SARS-CoV-2–specific clones that are first elicited early after infection and maintained up to 1 year, while increasing their binding and neutralization breadth. Given the emergence of viral variants and successful deployment of vaccines, future studies should address how and if similar clones can be reactivated upon vaccination or reinfection.

## Methods

### Sampling.

The study cohort was recruited from hospitalized COVID-19^+^ patients at the Sahlgrenska University Hospital, Gothenburg, Sweden. Prepandemic controls were obtained from healthy donors seronegative by ELISA to S and N.

Whole-blood samples and serum samples of patients were drawn at admission and every 3 days during hospitalization. After discharge, samples were drawn every 3 months for up to 1 year. Whole-blood samples were collected in lithium heparin tubes, from which PBMCs were isolated with Lymphoprep (Stemcell Technologies) and stored in liquid nitrogen. Serum samples were collected in serum tubes and stored at –80°C (28, 29).

### Preparation of immune cell populations.

For scRNA-Seq, PBMCs were thawed and divided in 3 fractions, labeled with barcoded Hashtag Abs 1–6 (BioLegend; catalogs 394661, 394663, 394665, 394667, 394669, 394671), in order to facilitate later demultiplexing, and submitted to different downstream handling. The starting number of cells was approximately 10 × 10^6^ cells/tube. For the first group, 300,000 total PBMCs only underwent dead cell depletion (EasySep Dead Cell Removal [Annexin V] Kit; Stemcell Technologies). In the second group, B cells were isolated from 700,000 cells of the crude sample via negative selection (EasySep Human Pan-B Cell Enrichment Kit; Stemcell Technologies), prior to dead cell removal. Both fractions were then incubated with Human TruStain FcX (BioLegend, catalog 422302) before being stained with dCODE Dextramer–PE (Immunodex, product code dCXC) complexes containing SARS-CoV-2 N, S, or the RBD of SARS-CoV-2 S (Sino Biological; catalogs 40588-V27B-B, 40591-V27H-B, 40592-V27H-B). The remaining PBMC fraction was used for isolation of SARS-CoV-2–specific B cells using FACS.

### FACS.

To test the specificity of our approach, COVID-19^+^ and prepandemic controls were stained with dCODE Dextramer–PE complexes, as well as complexes of SARS-CoV-2 S and N coupled with fluorophore-streptavidin conjugates (APC, BioLegend, catalog 405207). Following addition of anti–human CD19 APC-H7 (BD Biosciences, catalog 115530), IgD BUV395 (BD Biosciences, catalog 563813), and LIVE/DEAD Fixable Aqua Dead Cell Stain (Thermo Fisher Scientific, catalog L34966), cells were acquired on BDFACSAria Fusion X20.

For sorting of SARS-CoV-2–specific B cells, PBMCs were stained with TotalSeq anti–human Hashtag Antibody and Human TruStain FcX (BioLegend), similarly to the other 2 fractions. Cells were subsequently stained with dCODE Dextramer–PE complexes, as well as complexes of SARS-CoV-2 S or N coupled with fluorophore-streptavidin conjugates (APC and BV421, BioLegend, catalog 405225) in order to reduce background binding. Following addition of anti–human CD19 APC-H7, IgD BUV395, and LIVE/DEAD Fixable Aqua Dead Cell Stain, cells were sorted using a BDFACSAria Fusion. The whole sample was sorted.

### scRNA-Seq.

After being processed, the 3 immune cell population fractions were stained with TotalSeq-C Human Universal Cocktail, V1.0 (BioLegend, catalog 399905), as well as Total-Seq anti–human CD72, IgG Fc, CD197 (CCR7), CD45RB, CD193 (CCR3), TCR, LAIR1 (CD395), and CD366 (Tim-3) (BioLegend, catalogs 316211, 410727, 353251, 310211, 310733, 331231, 342807, and 345049, respectively). Cells were pooled and washed using the Laminar Wash Mini system (Curiox Biosystems) before using the Chromium Next GEM Single Cell 5’ Kit v2 and Chromium Next GEM Chip K Single Cell Kit (10X Genomics).

Libraries were created using the Library Construction Kit, 5’Feature Barcode Kit, Chromium Single Cell Human BCR Amplification Kit, Dual Index Kit TT Set A (PN-1000215), and Dual Index Kit TN Set A (PN-1000250) (10X Genomics).Their quality and quantity was assessed using the Agilent Tapestation system and the Qubit Fluorometer (Invitrogen), before they were sent for sequencing on Illumina NovaSeq 6000 as per the instructions provided in 10X Genomics user guide. Sequencing was performed by SNP&SEQ Technology Platform, Science for Life Laboratory (Uppsala Biomedical Centre, Uppsala University, Sweden).

### Data analysis.

Raw fastq files were processed through the 10X cellranger pipeline using the multicommand and default parameters with reference genome GRCh38-2020-A. Raw UMI count matrices generated from the cellranger 10X pipeline were loaded and merged into a single Seurat object. Cells were discarded if they met any of the following criteria: percentage of mitochondrial counts > 15%, number of unique features or total counts was in the bottom or top 0.5% of all cells, and number of unique features < 50. Red cells were discarded by filtering on *HBB*, *HBD*, *HBA1*, *HBA2*, *HBM*, and *HBQ1* expression.

Gene counts were normalized to the same total counts per cell and were natural log transformed (after the addition of a pseudocount of 1). The normalized counts in each cell were mean-centered and scaled by their SD, and the following variables were regressed out: number of features, percentage of mitochondrial counts, and the difference between the G2M and S phase scores. Surface protein and antigen probe were normalized by a centered log-ratio (CLR) normalization.

Data integration across cells originating from different samples was done using the Anchor method within Seurat. To integrate surface protein data and transcriptomic data, the FindMultiModalNeighbors function in Seurat was used to construct a WNN graph. For each cell, the nearest neighbors based on a weighted combination of 2 modalities was identified and used as input for dimensional reductions. Selection of the number of components for the nearest-neighbor network computation was based on their visualization in an elbow plot. UMAP (65) was performed for the spatial visualization of the single-cell data set after features and cells were clustered using the Louvain algorithm.

For CITE-Seq, we took advantage of 7 internal isotype controls included in the mixture (MouseIgG1, MouseIgG2a, MouseIgG2b, RatIgG2b, RatIgG1, RatIgG2a, and HamsterIgG) and performed QC using the scater and DropletUtils packages (66, 67). Briefly, the isotype controls were used as a measure of nonspecific protein aggregates and as a measure to assess the specificity of our protein labels.

Protein binding reads were normalized using the CLR function for each sample. To define probe-binding cells, we use the HTODemux function in Seurat. All positive cells were subsequently examined, and cells positive only for only 1 probe were assigned the corresponding specificity.

Cells that did not have an associated protein barcode or that had multiple barcodes were defined as nonbinding. RBD binding cells were defined as cells barcoded with both S and RBD. Interestingly, there was a very clear population of cells just having RBD barcodes assigned; given that we could not confidently assign those cells a clear specificity, we defined them as nonbinders. Therefore, cells defined as S specific included only non-RBD epitopes, while RBD cells were S binders with RBD specificity.

### BCR-Seq data processing.

The BCR-Seq data was processed using the Immcantation toolbox (v4.0.0) using the IgBLAST and IMGT germline sequence databases, with default parameter values unless otherwise noted. The IgBLAST database was used to assign V(D)J gene annotations to the BCR FASTA files for each sample using the Change-O package (68), resulting in a matrix containing sequence alignment information for each sample for both light- and heavy-chain sequences.

BCR-Seq database files associated with the same individual were combined and processed to infer the genotype using the TIgGER package (69), as well as to correct allele calls based on the inferred genotype. The SHazaM package (68) was used to evaluate sequence similarities based on their Hamming distance and estimate the distance threshold separating clonally related from unrelated sequences. For each patient, Ig sequences were assigned to clones using the hierarchicalClones function in Scoper (70), where the distance threshold was set to the corresponding value predicted with SHazaM in the previous step. Clonal assignment was done based on both heavy and light chain, and cells with multiple heavy or light chains were excluded. Germline sequences were generated for each patient using the genotyped sequences (FASTA files) obtained using TIgGER (69). BCR mutation frequencies were then estimated using SHazaM. The BCR-Seq data, clone assignments, and estimated mutation frequencies were integrated with the scRNA-Seq data by aligning and merging the data with the metadata slot in the processed RNA-Seq Seurat object.

### Differential gene expression analysis.

Differentially expressed genes between different clusters were identified using the FindAllMarkers function from Seurat using default settings (Wilcoxon test and Bonferroni *P* value correction). Significant genes with average log fold change > 0.25, and expressed in > 25% of cells in that group were ranked according to fold change. For comparison between 2 groups, the FindMarkers function was used, and all genes were represented on a VolcanoPlot.

### GO analysis.

Differentially expressed genes between 2 populations were identified as above and analyzed using the ClueGo plugin in Cytoscape (71, 72). Upregulated genes for each population were analyzed using the GOBiological and GOImmune terms with minimum 4 genes and 4% settings. Only pathways with *P* < 0.001 were considered. Common pathways were consolidated by clueGO. Pathways were exported and plotted.

### Selection and expression of mAbs.

Clonal families present at multiple time points were filtered, and sequences were manually inspected. Abs were selected based on binding characteristics, cell type, mutation rate, and isotype. Both heavy and light chains were synthesized, and mAbs were expressed as IgG1 in small scale by Biointron. After testing, further production was carried out in-house as follows. To express recombinant Abs, plasmids encoding corresponding heavy and light chains were mixed in equal ratio. Transfection of Expi293 cells was carried out by ExpiFectamine 293 Transfection Kit (Thermo Fisher Scientific) according to the manufacturer instruction. After 4–7 days, supernatants were collected and filtered. Purification of the immunoglobulins was carried out by Akta Start System (GE Healthcare) using protein G column. Elution of bound Abs was done by 0.1M glycine buffer (pH 2.7). To neutralize the low pH of the eluting solution, collection tubes contained 100 µL of 1M Tris buffer (pH 9.0). Ab-containing eluates were concentrated using VivaSpin columns with a 30 kDa cut-off. Tubes were spun at 3,500*g* at 4°C until the Abs reached a concentration of 1 mg/mL. Estimation of Ab concentration was done by NanoDrop (Thermo Fisher Scientific) equipment.

### Electrochemiluminescence assay.

Serum samples were analyzed for IgG, IgA, and IgM Abs binding to SARS-CoV-2 proteins. Additionally, sequenced BCR repertoires of the patients were used to design human IgG1 mAbs (produced by Biointron).

Binding to a panel of respiratory viruses was analyzed using a multiplex electrochemiluminescence assay including SARS-CoV-2 S, RBD, NTD, and N antigens, as well as SARS-CoV-1, MERS, OC43, and HKU1 S antigens and Influenza H3 antigen (V-PLEX COVID-19 Coronavirus Panel 1 [IgG, IgM, IgA] Kit, Meso Scale Discovery). SARS-CoV-2–specific Abs were further analyzed for binding to S antigen of several SARS-CoV-2 variants (V-PLEX SARS-CoV-2 Panel 23 [IgG] Kit, Meso Scale Discovery). For read-outs, we used the Meso Quickplex SQ 120 reader (Meso Scale Discovery).

Serum and plasma samples were analyzed at a 1:5,000 and/or 1:50,000 dilution, and mAbs were analyzed at 2–4 different concentrations, ranging from 0.01 μg/mL to 10 μg/mL. For analysis, binding signal was log transformed and normalized, using the normalize function in Prism (GraphPad), with the top binder set to 1.

### ELISA.

Microlon medium-binding half-well ELISA plates (Greiner Biotech) were coated overnight at 4°C with recombinant SARS-CoV-2 N (1 μg/mL), SARS-CoV-2 S, SARS-CoV-2 S_RBD, or IAV HA H3 Brisbane (all 2 μg/mL), in 25 μL PBS. Plates were blocked with 25 μL PBS/2% BSA for 1 hours at room temperature (RT). After 3 washes with PBS + 0.05% Tween-20 (PBST) using a BioTek 405 LS Plate Washer, plates were incubated with 3-fold dilutions of mAbs starting from 30 μg/mL in PBST for 1 hour at RT. After 3 washes, plates were incubated with 25 μL of anti–human IgG (Southern Biotech, catalog 2048-05) diluted 1:6,000 for 1 hour at RT. After 3 washes, plates were developed for 5 minutes using 1-step Ultra TMB (Thermo Fisher Scientific) and halted with 2M H_2_SO_4_. Plates were read at 450nm with a TECAN Plate reader.

### Pseudotyped virus neutralization assays.

Pseudotyped virus neutralization assays were performed as previously described (46). Briefly, pseudotyped lentiviruses displaying spikes (with C-terminal truncations) from the ancestral variant ,or from variants of concern, and packaging a firefly luciferase reporter gene were generated by cotransfecting HEK293T cells using Lipofectamine 3000 (Invitrogen) according to manufacturer protocols. Pseudotyped virions standardized to an input producing ~100,000 RLUs were incubated with serial dilutions of recombinant Abs for 60 minutes at 37°C prior to the addition of ~15,000 HEK293T-ACE2 cells and incubation for 48 hours; luminescence was measured using Bright-Glo (Promega) on a GM-2000 luminometer (Promega).

### Data availability.

The processed scRNA-Seq data reported in this paper are available in the ArrayExpress database (http://www.ebi.ac.uk/arrayexpress) under accession no. E-MTAB-12392. All codes supporting the current study are available from the corresponding author, upon request.

### Statistics.

For analysis of single-cell data, relevant statistical analyses are indicated in the respective method section. For comparisons of immune cell proportion, ordinary 1-way ANOVA with post hoc Tukey correction for multiple comparisons was used. Outliers were identified using Grubbs’ test. All statistical comparisons were performed using the rstatix package in R.

### Study approval.

The study was approved by the Swedish Ethics Review Authority (registration no. 2020–01771). Written informed consent was received from all patients, prior to participation

## Author contributions

Conceptualization was contributed by MB and DA; methodology was contributed by LS, HA, AE, NRM, DJS, IVS, BM, AL, MB, and DA; formal analysis was contributed by LS, HA, AE, DJS, AL, MB, and DA; investigation was contributed by LS, HA, AE, NRM, PI, IVS, and DJS; data curation was contributed by DA; resources were contributed by SL, IVS, NM, LMA, EM, MG, and BM; patient inclusion and sampling were contributed by LMA and MG; project administration was contributed by AL, MB, and DA; supervision was contributed by BM, AL, MB, and DA; funding acquisition was contributed by AL, BM, MB, DA, and MG; writing of the original draft was contributed by DA; review and editing of the manuscript were contributed by LS, EM, HA, AE, NRM, DJS, SL, PI, IVS, NM, LMA, MG, BM, AL, MB, and DA.

## Supplementary Material

Supplemental data

Supplemental table 1

## Figures and Tables

**Table 1:**
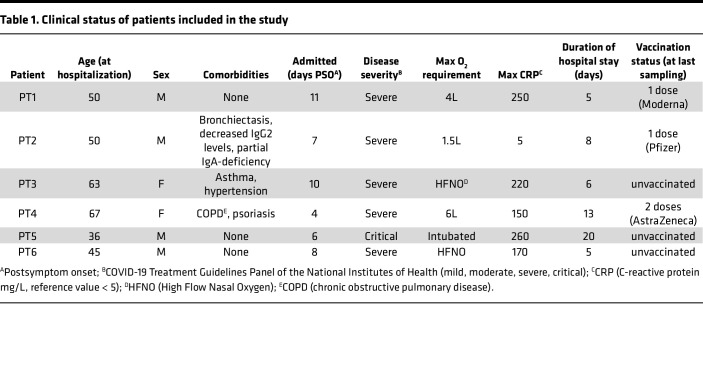
Clinical status of patients included in the study

## Version 1 11/29/2022

In-Press Preview

## Version 2 01/10/2023

Electronic publication
