# Supplementary material for: Longitudinal single-cell analysis of SARS-CoV-2–reactive B cells uncovers persistence of early-formed, antigen-specific clones
Source: JCI Insight. 2023 Jan 10;8(1):e165299. doi: 10.1172/jci.insight.165299 (PMC9870078; doi:10.1172/jci.insight.165299)
Supplement: Supplemental data [file jciinsight-8-165299-s211.pdf]

Longitudinal single-cell analysis of SARS-CoV-2-reactive B cells uncovers persistence of early-formed, antigen specific clones

Lydia Scharf[1], Hannes Axelsson[1], Aikaterini Emmanouilidi[1], Nimitha R. Mathew[1], Daniel J Sheward[2], Susannah Leach[1,3], Pauline Isakson[4], Ilya V. Smirnov[1], Emelie Marklund[5,6], Nicolae Miron[4], Lars-Magnus Andersson[5,6], Magnus Gisslén[5,6], Ben Murrell[2], Anna Lundgren[1,4], Mats Bemark[1,4,*], Davide Angeletti[1,*]

[1]Department of Microbiology and Immunology, Institute of Biomedicine, University of Gothenburg, Gothenburg, Sweden

[2]Department of Microbiology, Tumor and Cell Biology, Karolinska Institutet, Stockholm, Sweden

[3]Department of Clinical Pharmacology, Sahlgrenska University Hospital, Gothenburg, Sweden

[4]Department of Clinical Immunology and Transfusion Medicine, Sahlgrenska University Hospital, Region Västra Götaland, Gothenburg, Sweden

[5]Department of Infectious Diseases, Sahlgrenska University Hospital, Region Västra Götaland, Gothenburg, Sweden

[6]Department of Infectious Diseases, Institute of Biomedicine, University of Gothenburg, Gothenburg, Sweden

LS, HA and AE contributed equally to the study

[*]**Correspondence:**
Davide Angeletti, Department of Microbiology and Immunology, Institute of Biomedicine, Medicinaregatan 7A, 413 90 Gothenburg, Sweden. Phone: +46-(0)31-786 6324. Email: davide.angeletti@gu.se
Or
Mats Bemark, Department of Microbiology and Immunology, Institute of Biomedicine, Medicinaregatan 7A, 413 90 Gothenburg, Sweden. Phone: +46-(0)70-323 7043. Email: mats.bemark@immuno.gu.se

The authors have declared that no conflict of interest exists.

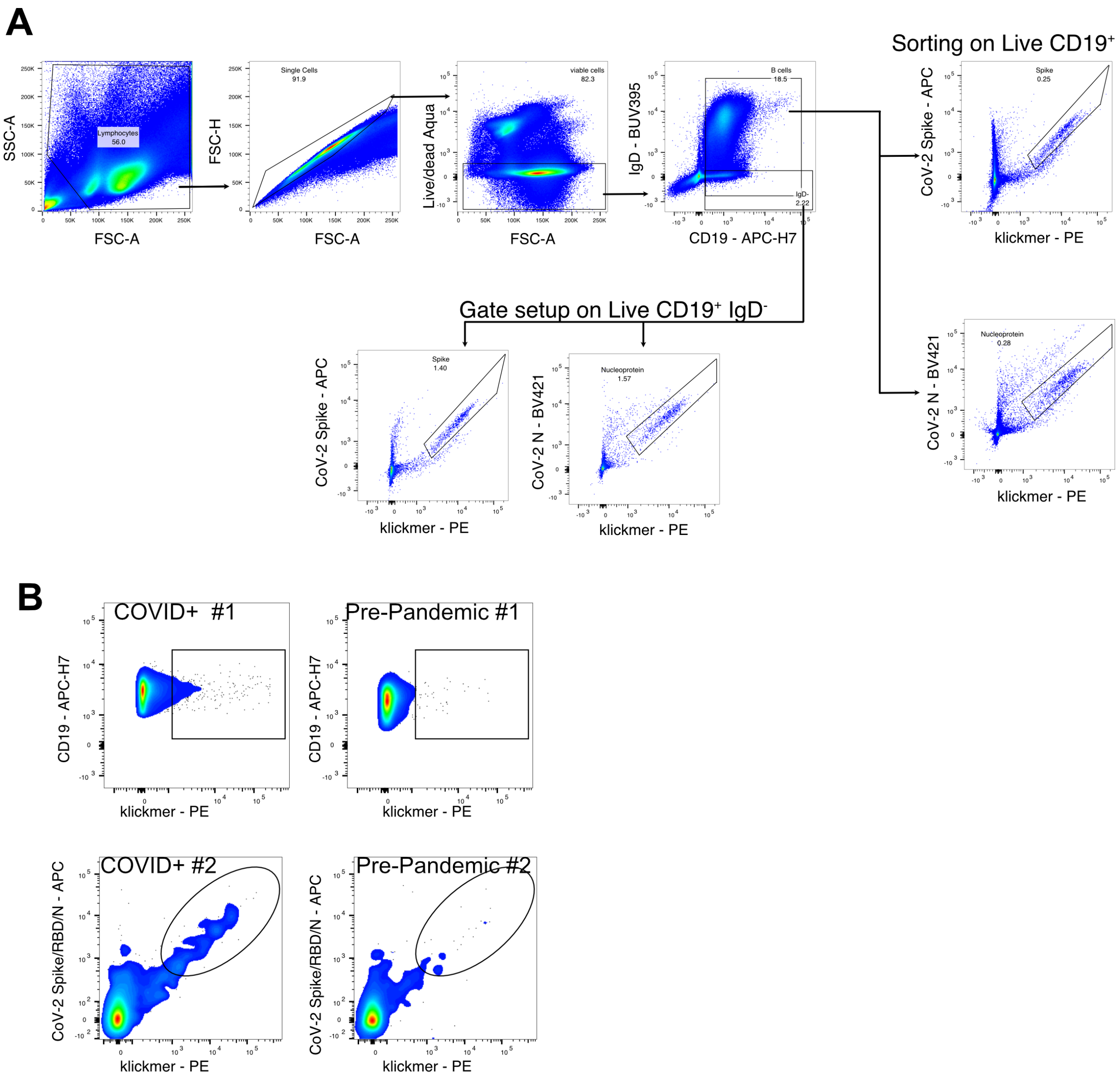

Supplementary Figure 1. A) Representative sorting strategy. Cells were sorted as Live, CD19+, Klickmer+ and either N+ or S+. For more accurate gating, sorting gates were drawn on IgD- population. B) To assess specificity, pre-pandemic controls were tested for their binding to Klickmer-PE (top, gated on Live, CD19+ IgD+) and Klickmer-PE and S-,RBD-, N-APC (bottom, gated on Live, CD19+ IgD+).

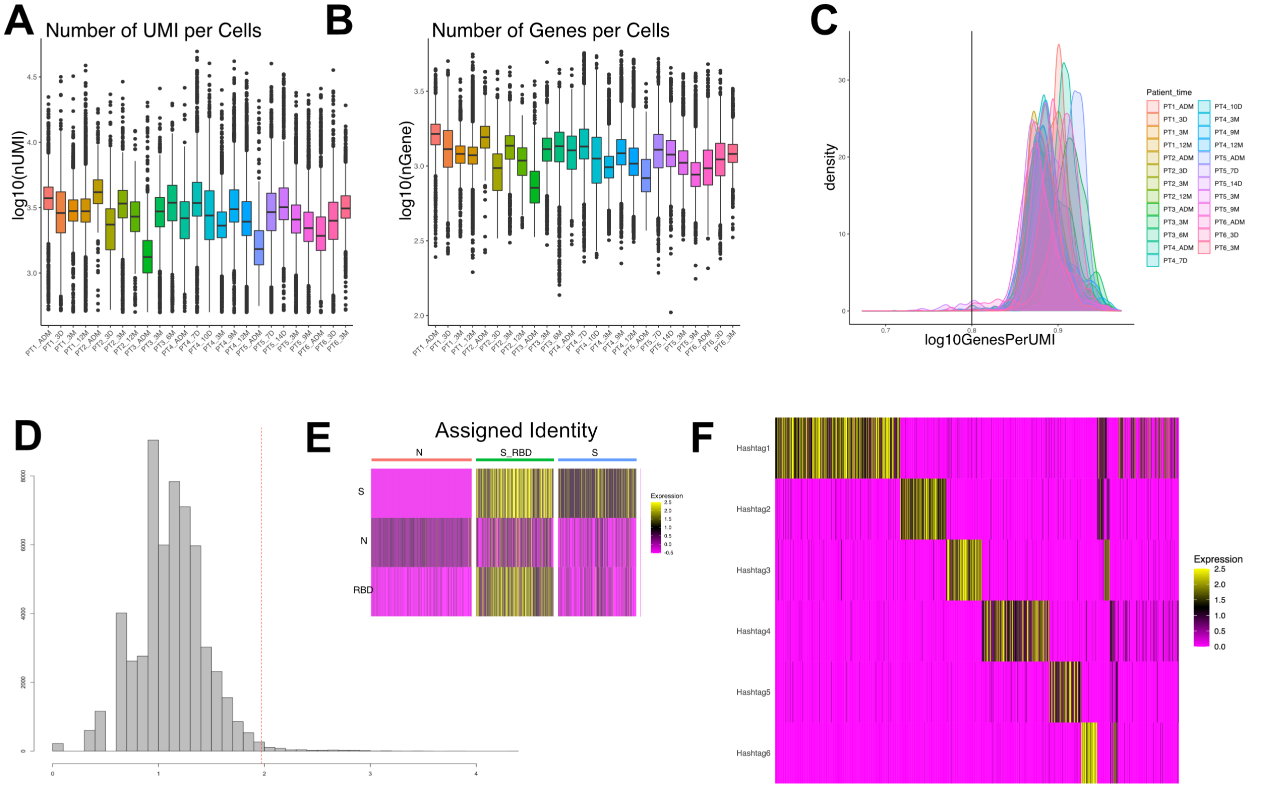

Supplementary Figure 2. Quality measures of single cell data. A) Number of UMI per cell, divided by sample. B) Number of genes per cell, divided by sample. C) Histogram showing cell complexity per sample, with the red vertical bar indicating the threshold of 0.8. D) Heatmap of CLR of Hashtags. D) Seven isotype controls were included in the CITE-Seq panel (MouseIgG1, MouseIgG2a, MouseIgG2b, RatIgG2b, RatIgG1, RatIgG2a, HamsterIgG). The coverage of these control Abs serves as a measure of non-specific binding in each cell, most notably protein aggregates. Red vertical line is the threshold for aggregates, showing very few cells (0.5% of cells). E) Heatmap of centered log ratio transformed counts (CLR) of barcoded PE-Dextramers showing specificity of the assign protein Identity. The graph considers only cells with assigned Protein Binding. F) Heatmap showing CLR of Hashtags.

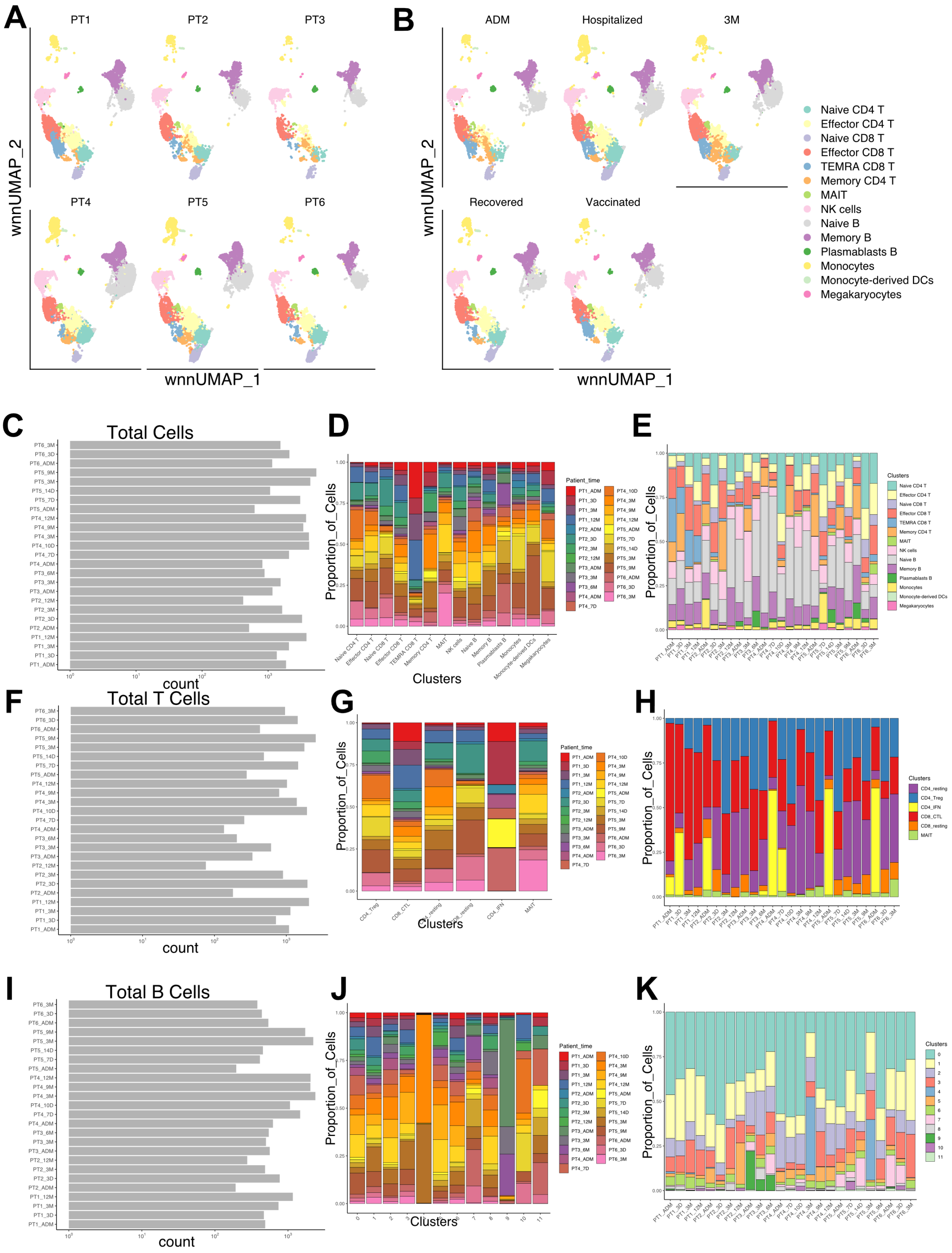

Supplementary Figure 3. A) UMAP plot as in Fig 1B but split according to patient. B) UMAP plot as in Fig 1B but split according to sampling time. Total number of cells (A), T cells (F) and B cells (I) obtained from each patient and time point. The graph reveals substantial homogeneity among total and B cells. Contribution from each sample to the total number of cells of each cluster for all cells (D), T cells (G) and B cells (J). Proportion of cells from each cluster, divided by patient and time point for all cells (E), T cells (H) and B cells (K).

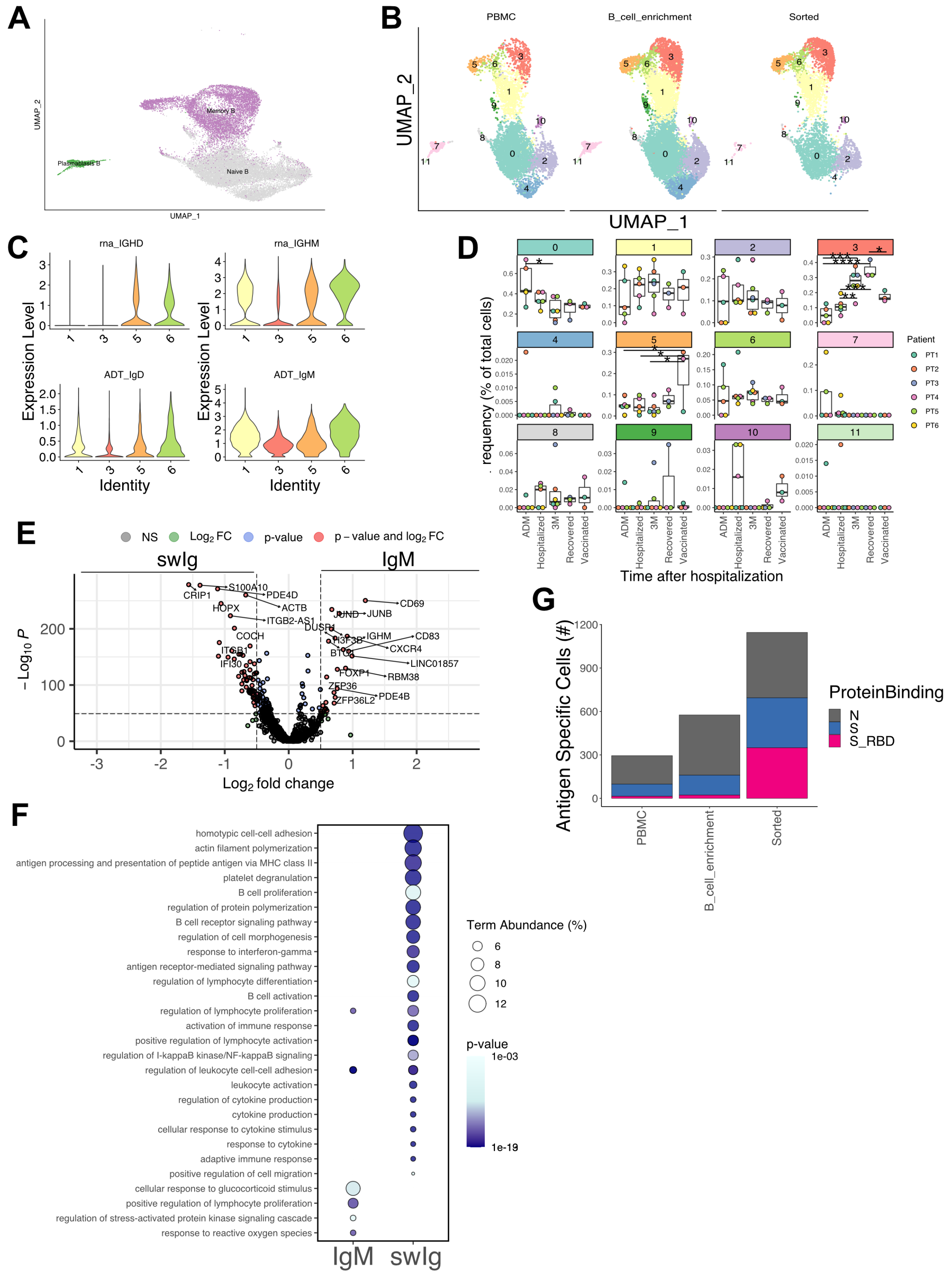
A
UMAP_2
UMAP_1
Memory B
Naive B
Plasmablasts B
B
PBMC
B_cell_enrichment
Sorted
UMAP_2
UMAP_1
C
rna_IGHD
rna_IGHM
ADT_IgD
ADT_IgM
Expression Level
Identity
D
Patient
PT1
PT2
PT3
PT4
PT5
PT6
ADM
Hospitalized
3M
Recovered
Vaccinated
Time after hospitalization
E
NS
Log2 FC
p-value
p – value and log2 FC
swIg
IgM
– Log10 P
Log2 fold change
S100A10
CRIP1
PDE4D
HOPX
ACTB
ITGB2-AS1
COCH
ITGB1
IFI30
CD69
JUND
JUNB
DUSP1
IGHM
CD83
PI3F3B
CXCR4
BTG1
LINC01857
FOXP1
RBM38
ZFP36
ZFP36L2
PDE4B
F
homotypic cell-cell adhesion
actin filament polymerization
antigen processing and presentation of peptide antigen via MHC class II
platelet degranulation
B cell proliferation
regulation of protein polymerization
B cell receptor signaling pathway
regulation of cell morphogenesis
response to interferon-gamma
antigen receptor-mediated signaling pathway
regulation of lymphocyte differentiation
B cell activation
regulation of lymphocyte proliferation
activation of immune response
positive regulation of lymphocyte activation
regulation of I-kappaB kinase/NF-kappaB signaling
regulation of leukocyte cell-cell adhesion
leukocyte activation
regulation of cytokine production
cytokine production
cellular response to cytokine stimulus
response to cytokine
adaptive immune response
positive regulation of cell migration
cellular response to glucocorticoid stimulus
positive regulation of lymphocyte proliferation
regulation of stress-activated protein kinase signaling cascade
response to reactive oxygen species
IgM
swIg
Term Abundance (%)
6
8
10
12
p-value
1e-03
1e-19
G
Antigen Specific Cells (#)
ProteinBinding
N
S
S_RBD
PBMC
B_cell_enrichment
Sorted

Supplementary Figure 4. A) UMAP plot as in Fig 3A but with grouping defined as in Fig 1. B) UMAP plot as in Fig 3A but split according to origin of sample. C) Violin plot showing expression of IgM and IgD genes (rna) or proteins (ADT) within the MBC clusters. “ADT” indicates surface protein expression while “rna” shows transcript expression. D) Frequency for each of the identified B cell clusters, indicated for each time of sampling and patient. Only cells belonging to the antigen-sorted pool were considered for this analysis. Multiple comparisons were performed using one-way ANOVA with Tukey´s multiple comparison test. * = $p<0.05$ E) Volcano Plot showing differential expression of genes between switched memory cells (clusters 3 and 5) and IgM-MBC (cluster 1). F) Differentially expressed genes between IgM and swIg were analyzed using ClueGo and significant pathways plotted. Size of the dot indicates the % of term in the GO category, while color intensity represents the *p* value. G) Number of protein binding cells for each of the populations purified. Clear enrichment is present in the sorted cell population

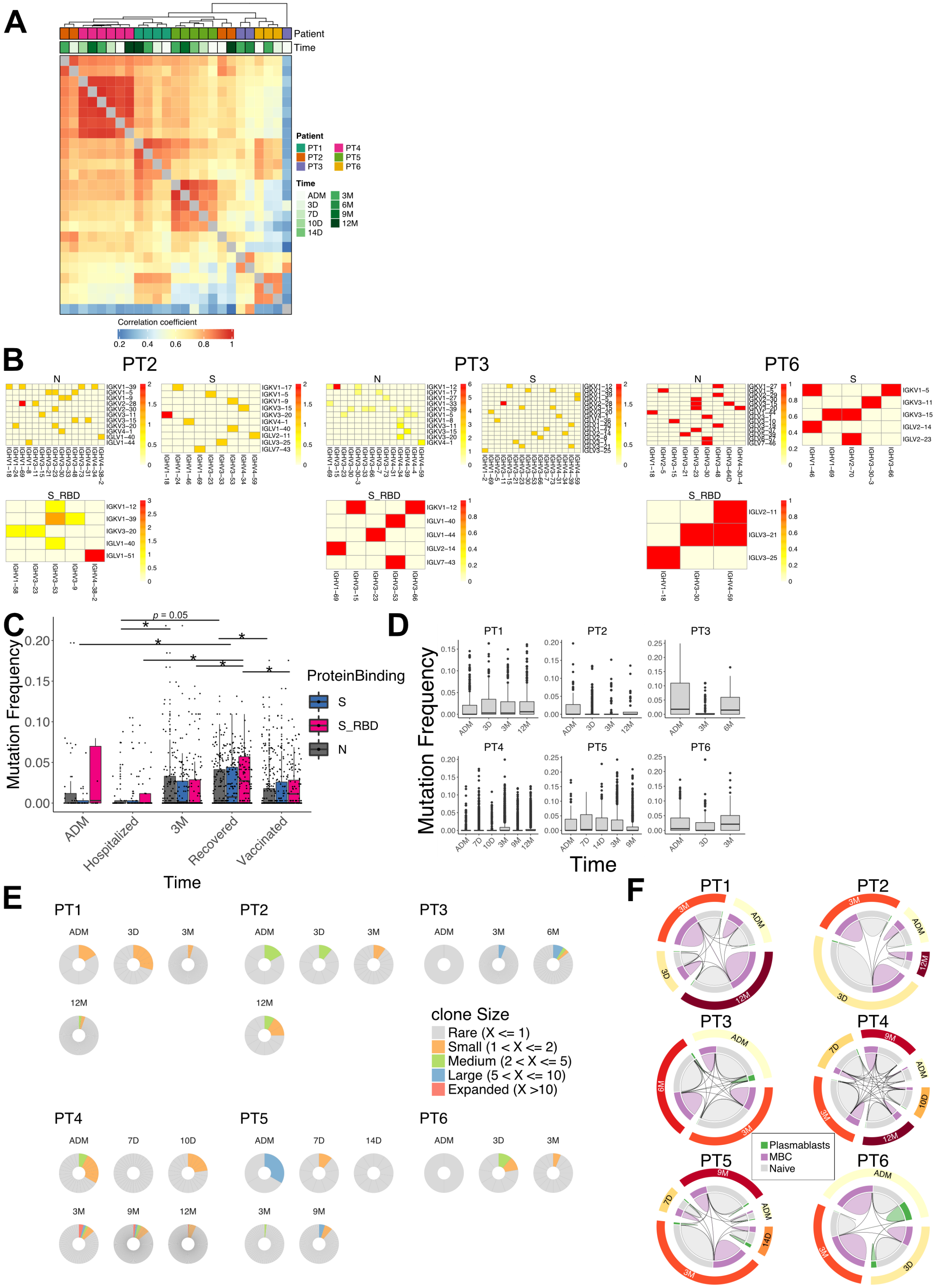

A
Patient
Time
Patient
PT1
PT2
PT3
PT4
PT5
PT6
Time
ADM
3D
7D
10D
14D
3M
6M
9M
12M
Correlation coefficient
0.2
0.4
0.6
0.8
1
B
PT2
PT3
PT6
N
S
S_RBD
C
Mutation Frequency
ProteinBinding
S
S_RBD
N
p = 0.05
ADM
Hospitalized
3M
Recovered
Vaccinated
Time
D
Mutation Frequency
PT1
PT2
PT3
PT4
PT5
PT6
Time
E
PT1
PT2
PT3
PT4
PT5
PT6
clone Size
Rare (X <= 1)
Small (1 < X <= 2)
Medium (2 < X <= 5)
Large (5 < X <= 10)
Expanded (X >10)
F
PT1
PT2
PT3
PT4
PT5
PT6
Plasmablasts
MBC
Naive

Supplementary Figure 5. A) Hierarchical clustering of Pearson's correlation of the V gene repertoire. Each tile represents the correlation of the V gene repertoire. Color intensity indicates correlation strength. B) Heatmaps showing the frequency of each patient´s heavy- and light-chain gene pairings for B cells binding the indicated antigens. C) Graph showing Vh gene mutation frequency for all patients combined, but divided by time of sampling and antigen binding. Data are presented as median and interquartile range. D) Graph showing Vh gene mutation frequency divided by patient and time of sampling for non-binding cells. Data are presented as median and interquartile range. Multiple comparisons were performed using two-way ANOVA with Bonferroni correction for multiple comparisons. * = $p<0.05$ E) Pie chart showing B cell clonal expansion divided by patient and time point, for clonal families with at least 2 members. B cells were binned into rare clones (1 member), small (2 members), medium (between 3 and 5 members), large (between 6 and 10 members) and expanded (over 11 members). F) Circos plot showing clonal relationship within each patient at different sampling times. Connecting lines indicate shared clones and the size of the circle and connector is proportional to the repertoire space occupied. Outer circle indicates sample time while inner circle and connectors are colored depending on cell type.

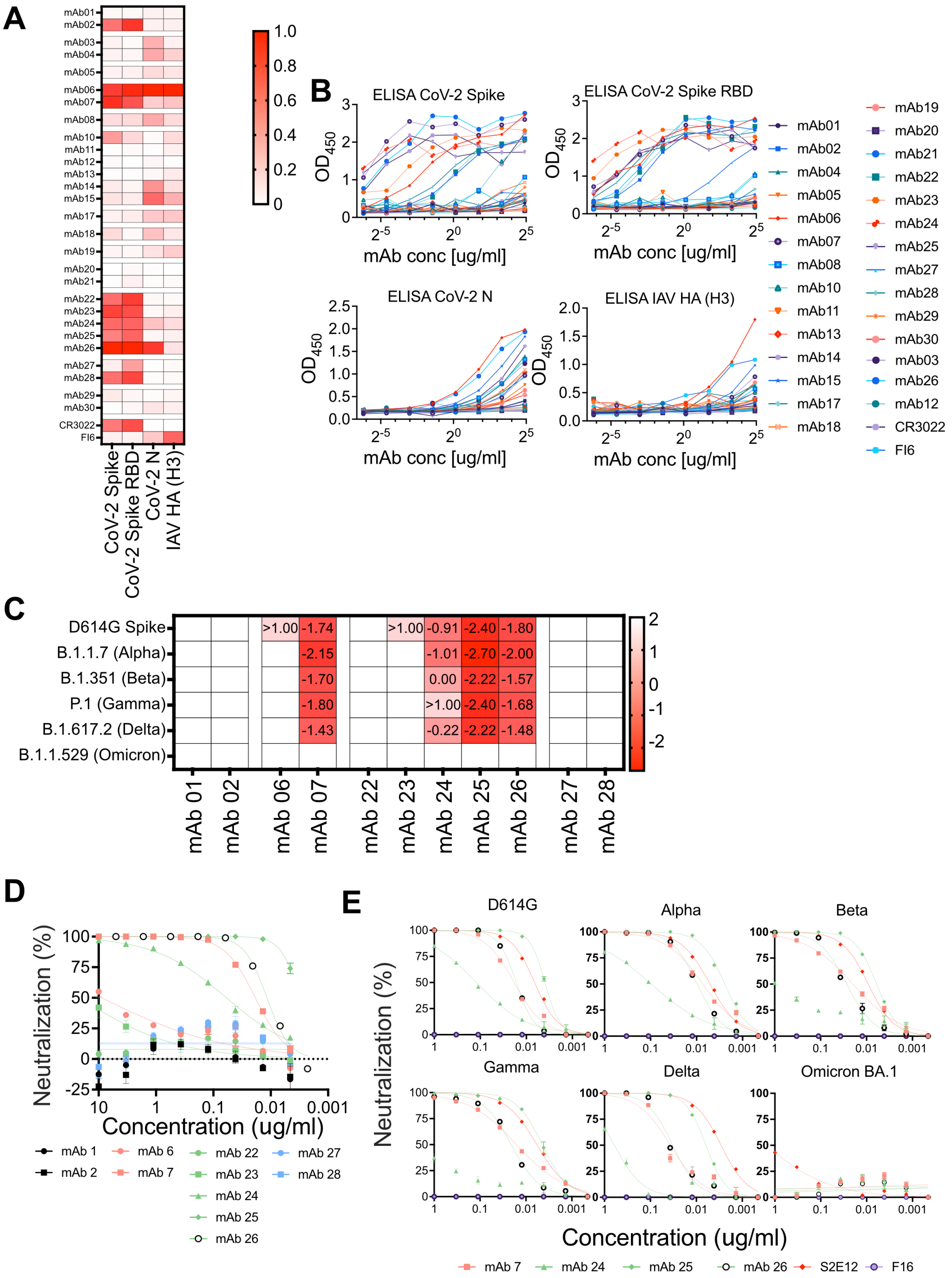

Supplementary Figure 6. A) Heatmap generated by ELISA binding to CoV-2 or influenza antigens. Color intensity indicates normalized Area Under the curve (AUC). B) ELISA curves used to generate the data in A. C) mAbs neutralization of pseudotyped lentivirus expressing S proteins from SARS-CoV2 viruses with Table showing IC50 of selected mAbs to viruses, as indicated on the left. Color intensity indicates the log IC50 value. Value >1 indicates detectable but low neutralization while white boxes denote no neutralization. D-E) Pseudovirus neutralization curves used to generate the data in A. Error bar depicts SD of at least 3 technical replicates
